# Postoperative Pain Following Gynecology Oncological Surgery: A Systematic Review by Tumor Site

**DOI:** 10.3390/cancers17162718

**Published:** 2025-08-21

**Authors:** Selina Chiu, Helen Staley, Xiaoxi Zhang, Anita Mitra, Flavia Sorbi, James Richard Smith, Joseph Yazbek, Sadaf Ghaem-Maghami, Sanooj Soni, Christina Fotopoulou, Srdjan Saso

**Affiliations:** 1 Division of Surgery and Cancer, Imperial College London, London W12 0NN, UK; 2 Department of Gynaecology Oncology, Imperial College Healthcare NHS Trust, London W12 0HS, UK; 3 Department of Gynaecology, Chelsea and Westminster Hospital NHS Foundation Trust, London SW10 9NH, UK; 4 Department of Anaesthetics, Imperial College Healthcare NHS Trust, London W2 1NY, UK; 5 Department of Gynaecology Oncology, West Hertfordshire Teaching Hospitals NHS Trust, Watford WD18 0HB, UK; 6 Department of Obstetrics and Gynaecology, University of Florence, 50134 Florence, Italy

**Keywords:** postoperative pain, acute pain, gynecologic cancer surgery, pain management, analgesia, complementary medicine

## Abstract

**Introduction:** Postoperative pain management is complex and crucial in major gynecology oncological surgery. Currently, there is no well-defined standardized approach, resulting in significant variability in practices worldwide. This systematic review evaluates the effectiveness of analgesic strategies used postoperatively in gynecological cancer surgery. **Methods:** A systematic review was conducted from inception to June 26th 2024 to identify all randomized controlled trials (RCTs) assessing pain management following any surgery for gynecological cancer. This was performed on the CENTRAL, PubMed, Embase, and MEDLINE databases. **Results:** A total of 46 RCTs met the inclusion criteria. Of these 5316 patients, 1844 patients had cervical cancer, 99 had endometrial cancer, and 158 had ovarian cancer. The remaining 3215 participants had unspecified gynecological cancers or benign pathology. No studies focused on postoperative analgesia for vulval cancer. A meta-analysis was not feasible due to heterogeneity in study design, analgesic interventions (i.e., opioids, local anesthetics, paracetamol, NSAIDs, and holistic and complementary therapies), and multiple routes of administration (i.e., oral, parenteral, regional, neuraxial, local infiltration, intraperitoneal, intramuscular, patient-controlled, topical, and rectal). No single analgesic modality demonstrated clear superiority. The median Jadad score for methodological quality of the included trials was 4. **Conclusions:** The limited cancer-specific RCTs and diversity of analgesia modalities utilized reflect the wide range of applications. Postoperative pain is multifactorial and cannot be adequately managed with a single agent. National and international guidelines should aim to establish a standardized framework for postoperative pain management in gynecological cancers, ensuring accessible, evidence-based care that enhances both short- and long-term patient quality of life.

## Simple Summary

Effective pain control after surgery for gynecological cancers is vital for patient recovery, yet current practices vary widely across different healthcare settings. This study reviewed and analyzed existing research to understand how pain is currently managed after these surgeries and to identify the most effective approaches. By examining 46 clinical trials involving over 5000 patients, the authors found that no single method worked best for everyone, but using a combination of treatments tended to improve pain control. The study highlights the need for clear and consistent guidelines to ensure all patients receive the most effective and personalized care after surgery. These findings aim to guide future research and support healthcare professionals in improving pain management for women undergoing gynecological cancer surgery.

## 1. Introduction

Postoperative analgesic management has evolved significantly over recent decades, with advances in the perception, definition, and measurement of surgery-related pain. Despite this progress, there is a lack of well-defined, standardized protocols tailored to specific surgery and cancer types. This variability results in suboptimal pain control, adversely impacting short- and long-term patients’ quality of life following major gynecology oncological surgery [1,2,3,4].

Poorly controlled postoperative pain is an independent risk factor for reduced patient satisfaction, prolonged recovery, higher readmission rates, development of chronic pain with increased overall surgical morbidity and mortality [5]. Effective postoperative pain management is crucial in patient recovery. However, uncontrolled postoperative pain remains a major problem affecting 85% of patients [6]. While numerous analgesic options exist and understanding of pain pathophysiology and perception has improved, there is no universally agreed pain management approach.

There has been great advancements underpinning the management of pain, utilizing different treatment modalities targeting specific aspects of the pain signaling pathway [7]. Numerous studies have demonstrated the effectiveness of a multimodal approach, combining systemic with regional analgesic techniques to reduce long-term opioid requirements [8]. The purpose of this systematic review is to evaluate these various analgesic options in their effectiveness according to tumor site.

## 2. Materials and Methods

### 2.1. Search Strategy

The systematic search followed Preferred Reporting Items for Systematic Reviews and Meta-Analyses (PRISMA) guidelines. This protocol was not registered in PROSPERO, as it was still under refinement during the initial phases of the study, and the decision was made to proceed without formal registration.

### 2.2. Literature Search

A comprehensive literature search was performed in the Cochrane Central Register of Controlled Trials (CENTRAL), PubMed, Embase, and MEDLINE databases for articles published from inception until 26 June 2024. The search strategy combined controlled vocabulary terms (e.g., MeSH) and keywords related to postoperative pain management (e.g., “analgesia,” “postoperative pain,” “pain relief”) and gynecological cancers (e.g., “ovarian cancer,” “cervical neoplasms,” “endometrial carcinoma,” “vulvar neoplasms”). Boolean operators and proximity searches were employed to enhance sensitivity and specificity. The detailed search strategies for each database are provided in Supplementary Material S1. Searches were limited to English-language publications.

### 2.3. Study Selection

All randomized controlled trials (RCT) investigating postoperative pain management following surgical treatment for gynecological cancers (cervical, endometrial, ovarian or vulval) were included. Eligible studies were required to assess pain severity using standardized and validated scoring systems, such as the Visual Analog Scale (VAS), Numeric Rating Scale (NRS), or comparable tools, which provide quantifiable measures of patient-reported pain.

Studies involving participants undergoing medical or radiological treatments for management of gynecological cancers were not included. Additional exclusion criteria included conference abstracts, commentaries, study protocols, non-randomized designs, preclinical research, secondary analyses of data from prior randomized controlled trials, and studies not published in English.

### 2.4. Data Extraction and Quality Assessment

**Figure 1:**
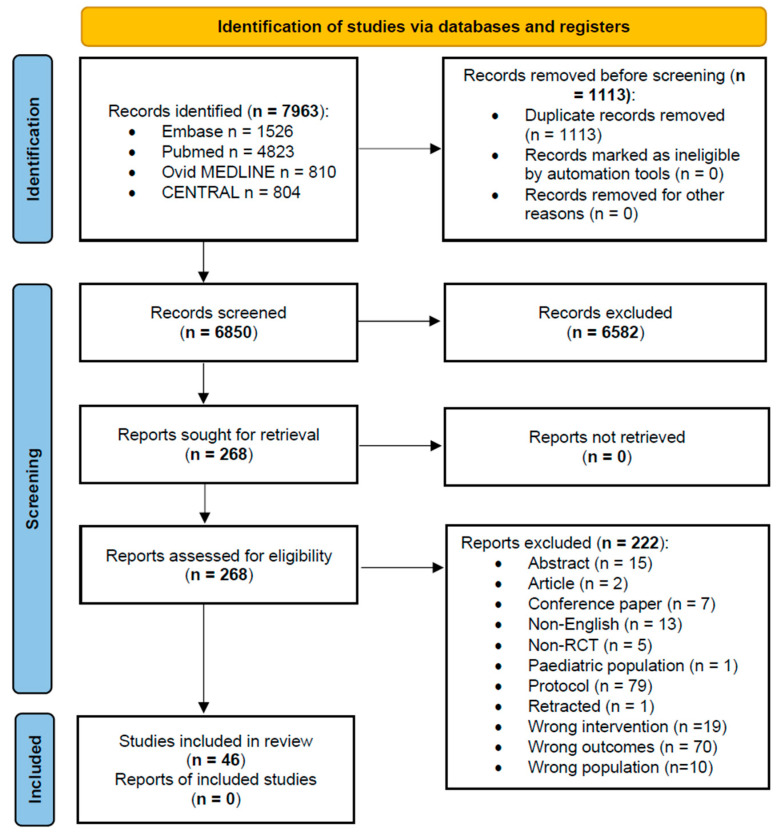
PRISMA flowchart of included studies.

Following removal of duplicate records, two independent reviewers (SC and HS) screened 6850 titles and abstracts, followed by full-text assessment for eligibility. Disagreements regarding study inclusion were resolved through consensus and when necessary, were adjudicated by a third reviewer (SS). An overview of the search results and screening process is summarized in the PRISMA flow diagram (Figure 1).

Data extraction was conducted using a pre-tested form within the Covidence platform (Covidence, Melbourne, Australia). The first author (SC) performed the initial data extraction for each included study, which was subsequently reviewed and verified by the second author (HS). Extracted data included publication year, country of origin, sample size, type of intervention, primary and secondary outcomes along with their corresponding measures, duration of follow-up, journal of publication, journal impact factor, and any reported commercial funding. Table 1 summarizes the main characteristics of the studies.

Two reviewers (SC and HS) independently assessed study methodological quality using the Jadad score and the Cochrane Collaboration’s risk of bias tool [54,55]. Where there were disagreements between reviewers, consensus was obtained after discussion with a third reviewer (SS).

### 2.5. Data Analysis

Data were analyzed according to tumor site. Studies that included participants with different types of gynecological cancers (e.g., ovarian, cervical, endometrial, cervical) but did not differentiate between pain analysis according to specific cancer types were categorized as generic cancers. RCTs that analyzed both benign and malignant gynecological surgical procedures, without reporting separate outcomes between the benign and malignant cases, were defined as generic (benign and cancer).

Authors were contacted in an attempt to attain data for tumor site-specific gynecological cancers in the studies that combined gynecological cancers with benign conditions. If site-specific data could not be provided, these studies were kept in the generic categories for analysis.

Statistical analysis was performed using SPSS version 25.0 (IBM, Armonk, NY, USA), with the median and interquartile ranges (IQR) used to summarize continuous non-parametric variables. Inter-rater reliability for study screening was assessed using Cohen’s kappa statistic to evaluate the level of agreement between reviewers.

Substantial clinical and methodological heterogeneity was observed across the included studies, including variability in patient populations, surgical procedures, intervention types, outcome measures, and timing of postoperative pain assessments. Differences in pain measurement tools and follow-up durations further contributed to this heterogeneity, rendering quantitative meta-analysis inappropriate. Consequently, a qualitative synthesis was conducted to narratively summarize and interpret the findings, emphasizing common patterns and notable differences among studies.

In accordance with the journal’s guidelines, we will provide our data for independent analysis by a selected team by the Editorial Team for the purposes of additional data analysis or for the reproducibility of this study in other centers if such is requested.

## 3. Results

Following exclusion of 1113 duplicates, 6850 titles and abstracts were screened. Two hundred and sixty-eight full texts were assessed for eligibility. After a further exclusion of 222 full texts, 46 RCTs were included in this systematic review. Inter-rater reliability was evaluated using Cohen’s kappa statistic, yielding κ = 0.653 for title and abstract screening and κ = 0.821 for full-text screening, indicating substantial to strong agreement.

The included studies were published between 2000 and 2024 (median year: 2020). The included trials involved 5316 participants of which 1844 had cervical cancer, 99 had endometrial cancer, and 158 had ovarian cancer. The remaining 3215 participants were unspecified gynecological cancers or benign pathology (Figure 1).

### 3.1. Cancer Types

There were 29 trials which investigated postoperative analgesia in gynecology cancer-specific operations, of which 11 trials were cervical cancer [8,9,10,11,12,13,14,15,16,17,18], 1 trial was endometrial cancer [19], 4 trials were ovarian cancer [20,21,22,23] and 13 trials assessed multiple gynecological cancers [24,25,26,27,28,29,30,31,32,33,34,35,36]. Seventeen trials included both gynecological cancers and benign pathology [37,38,39,40,41,42,43,44,45,46,47,48,49,50,51,52,53]. There were no studies investigating postoperative analgesia use for vulval cancer (Table 2).

### 3.2. Surgical Approach

Minimally invasive surgery (MIS) was analyzed in 11 trials (4 cervical; 1 endometrial; 2 generic cancer; 5 generic (benign and cancer) [8,13,15,17,19,34,35,37,39,42,51]. Seventeen trials reviewed open abdominal surgeries (1 cervical; 3 ovarian; 8 generic cancers; 5 generic (benign and cancer) [16,20,22,23,24,25,26,28,29,30,31,32,40,44,45,46,50]. Three trials assessed both minimally invasive surgery and open abdominal surgery (3 generic (benign and cancer) [38,48,52]. One study assessed the loop electrosurgical excision procedure (1 generic (benign and cancer) [41]. The surgical approach was not specified in 14 trials (6 cervical; 1 ovarian; 3 generic cancers; and 4 generic (benign and cancer)) [9,10,11,12,14,18,21,27,33,36,43,47,49,53].

### 3.3. Methods of Pain Assessment

Table 1 summarizes the included trials with the interventions investigated, methods of pain assessment, and monitoring duration interval. Thirty one trials used Visual Analog Scale (VAS) scores to assess pain [9,10,11,12,13,14,15,16,17,19,20,21,23,24,26,28,30,33,34,36,37,39,40,41,42,43,47,48,50,51,52], eleven trials used Numerical Rating Scale (NRS) [8,18,22,25,29,31,38,44,45,46,53], and four trials used other pain scales [27,32,35,49]. Seventeen trials were performed in China [8,9,10,11,12,13,14,15,16,17,18,19,34,35,38,42,47], five in USA [32,39,40,49,52], four in Turkey [33,36,41,43], and three in Egypt [20,24,46]. Two trials were performed in Germany [21,23], Iran [30,50], Japan [29,51], Sweden [22,44], and Thailand [26,37]. One trial was performed in Australia [28], Hong Kong [45], India [25], Italy [48], Republic of Korea [27], Poland [31], and Taiwan [53].

### 3.4. Types and Mode of Analgesia

Due to the heterogeneity of the trials, the results are presented in Table S2. Table 3 summarizes the types of analgesics and mode of administration in each study. The diversity across studies precluded meta-analysis and qualitative comparisons, highlighting the wide range of analgesic approaches, which limits generalizability.

Most studies (n = 37) included an opioid in at least one arm [8,9,12,13,15,16,17,19,21,22,23,25,26,27,28,29,30,31,32,33,34,36,37,38,39,40,42,43,44,45,46,47,49,50,51,52,53], while 23 studies involved non-steroidal anti-inflammatory drugs (NSAIDs) [8,9,12,13,16,19,26,27,28,31,33,35,36,37,39,40,42,44,45,47,49,51,52]. Seventeen trials utilized local anesthetics [8,10,20,21,22,23,24,25,28,29,30,39,40,41,44,46,51], 10 studies used paracetamol [25,31,36,39,44,45,49,50,51,52], and 5 explored holistic and complementary therapies [11,32,35,37,45].

Three studies included dexmedetomidine [27,34,46], 1 assessed magnesium with local anesthetics in a TAP block [24], 3 addressed psychological and empathetic care [11,14,18], and 3 explored lifestyle measures (diet and exercise) [18,35,48]. Six studies examined other drugs, including Metamizole, Duloxetine, Gabapentin, Ketamine, Opioid receptor antagonists, and Naloxone [7,15,31,35,50,53], while 2 evaluated abdominal binders [26,35].

Thirty-five studies used parenteral analgesia [8,10,12,13,15,16,17,19,21,22,23,26,27,28,29,30,31,33,34,35,36,38,39,40,41,42,43,44,45,46,47,49,50,52,53], 25 included patient-controlled analgesia (PCA) [8,12,15,16,17,22,23,25,26,27,28,29,32,33,34,36,38,40,42,43,45,47,49,52,53], and 9 involved oral medications [11,26,37,39,44,48,49,50,52]. Regional anesthesia was used in 6 studies [10,16,20,24,29,39], local anesthesia in 12 [21,22,23,25,28,29,30,36,40,43,44,46], and other delivery methods such as intraperitoneal [22], intramuscular [34], topical [41], and rectal [31,51] were also investigated.

### 3.5. Cervical Cancer

Two studies assessed the impact of nursing interventions, which significantly reduced pain levels [14,18]. One study reported reduced pain levels at 6, 24, 48 and 72 h with a structured care model incorporating pain-specific training, psychological support, and enhanced medication compliance [14]. Another study showed that an empathetic care model, which included training in emotional support, communication techniques, and interdisciplinary collaboration, significantly improved pain management [18].

NSAIDs were also shown to decrease postoperative pain compared to control groups [8,9,12,13]. One study analyzed the effects of continuous infusion, bolus, and patient-controlled opioid delivery, finding that both continuous and patient-controlled time-scheduled decremental oxycodone regimens significantly reduced pain at 1, 6, and 48 h, with the latter group using a lower cumulative dose compared to the other groups [17].

Two RCTs demonstrated that regional anesthesia with local anesthetics was an effective analgesic approach. Specifically, ilioinguinal, iliohypogastric, and erector spinae plane blocks (ESPB) provided effective pain relief, while the transverse abdominis plane block (TAPB) showed minimal improvement [10,16]. Additionally, one study reviewed various doses of ketamine and its enantiomers, all of which significantly improved postoperative pain at 1, 2, and 3 days compared to controls [15].

### 3.6. Endometrial Cancer

One study compared remifentanil and propofol bolus injections prior to extubation, finding no significant difference in VAS scores. However, remifentanil was associated with a shorter PACU stay [19].

### 3.7. Ovarian Cancer

A RCT found that ESPB significantly reduced postoperative pain at 0–30 min, 2–4 h, and 18 h compared to TAPB, leading to reduced tramadol use for rescue analgesia [20]. Two studies compared different local anesthetics in neuraxial blockades showed no significant differences in pain relief [21,23]. Another study evaluated intraperitoneal local anesthetic versus control with no significant improvement in analgesic efficacy [22].

### 3.8. Generic Cancer

One study showed that dexmedetomidine infusion significantly reduced postoperative pain during activity at 1 h but had no effect at rest or at other time points compared to control [37]. Another RCT showed no significant difference in analgesic effects between IV PCA Dexmedetomidine and Fentanyl [34].

Four studies examined neuraxial anesthesia for postoperative pain management in gynecologic-oncologic surgery, showing mixed results. The mean pain severity during first ambulation was significantly lower in the patient controlled epidural analgesia (PCEA) group compared to the IV PCA group (*p* < 0.001) [30]. Pain scores at rest at 24 h and during coughing at 12 and 24 h were significantly lower in the epidural group than the parenteral opioid group, though VAS scores were similar between the parenteral lidocaine and epidural groups [36]. Another study found continuous rectus sheath block (CRBS) significantly superior to continuous epidural analgesia (CEA) at 24 h post-surgery [29] and adding patient-controlled to CEA provided no additional benefit in pain relief [25].

In pain management strategies, one study identified that Metamizole (without Ketoprofen) was inferior to acetaminophen when combined with Morphine on day 0 postoperatively. However, a multimodal approach (Morphine, Acetaminophen, and Ketoprofen, or Morphine, Metamizole, and Ketoprofen) provided equivalent analgesia, regardless of whether acetaminophen or metamizole was used [31].

Another study demonstrated the addition of magnesium to TAP blocks significantly improved postoperative pain at all time points apart from at 8 h [24]. Two studies compared parenteral NSAIDs (Tenoxicam, Ketoprofen) with placebo showed no significant difference in pain scores [28,33]. However, the cumulative PCA-Tramadol consumption was significantly lower in Ketoprofen treated patients (*p* < 0.05) [33].

### 3.9. Generic (Benign and Cancer)

Three studies evaluated the analgesic effects of opioids. One compared opioid use to a non-opioid regimen (Dexmedetomidine and Lidocaine), with the latter showing significantly lower pain scores [46]. Two others compared different parenteral opioids, yielding conflicting results regarding postoperative pain relief [39,42]. Another study assessed the addition of nalbuphine to an opioid-based PCA compared to opioid PCA alone showing no significant difference at any time points [52].

Two studies examined NSAIDs following gynecologic-oncologic surgery. One found significantly lower pain scores at rest and during movement in the Parecoxib group compared to controls, reflecting reduced PCA morphine use postoperatively [47]. In contrast, another study found no difference in analgesic outcomes between oral Celecoxib and IV Ketorolac [51].

One study showed the addition of Duloxetine to routine postoperative pain management significantly decreased pain compared to placebo at 2–48 h postoperatively [49].

Seven studies investigated the impact of the route of administration on postoperative pain management. Bupivacaine with epinephrine in TAP blocks provided pain relief comparable to high-volume local port-site infiltration, with a significant benefit in the US-guided TAP block group at 2 h [40]. Thoracic PCEA morphine with bupivacaine was superior to parenteral morphine, significantly reducing pain scores both at rest and during coughing, with PCEA patients reporting less pain overall [41]. Three studies found no significant differences between neuraxial, parenteral, or oral morphine administration methods [43,44,48]. Sugihara et al. observed no overall significant difference in pain scores with the addition of levobupivacaine infiltration, though significant differences were noted at 2 h postoperatively in patients undergoing laparoscopic-assisted vaginal hysterectomy (*p* = 0.047) and laparoscopic hysterectomy (*p* = 0.007) [50]. Güngördük et al. found no significant difference in pain scores between local anesthetic (Lidocaine spray on the ectocervix followed by Bupivacaine submucosal injection) and general anesthesia for loop electrosurgical excision procedures [53].

### 3.10. Holistic and Complementary

Seven studies assessed holistic and complementary medicine. Hou et al. compared Chinese herbal treatment alone versus combined with psychological care in cervical cancer patients, finding no significant difference in acute postoperative pain on days 1 and 7 [11]. Ariyasriwatana et al. observed a significant reduction in pain with Curcumin capsules added to standard analgesia [37]. Similarly, low to medium-intensity walking exercises (at least 150 min per week before surgery) combined with routine nursing care resulted in lower pain scores beyond 24 h [35].

Utilization of an abdominal binder significantly reduced pain on days 1 and 2, but this effect was not sustained on day 3, with no overall change in pain scores from baseline to days 1 to 3 [26]. Postoperative massage or physiotone vibrational medicine, as well as acupuncture administered pre- and postoperatively (daily for up to five days), did not improve pain outcomes [32,45]. Palaia et al. also reported no significant difference between a low-residue and free diet starting three days before surgery [48].

### 3.11. Quality of Studies

Table 4 summarizes the primary and secondary outcomes assessed in each included trial. Table 5 summarizes our assessment of the quality of the included trials. Overall, the median Jadad score for methodological quality of the included trials was 4 (interquartile range (IQR) 2, minimum 1, maximum 5). The median impact score of the included journals was 2.741 (IQR 2.280, minimum 0.196, maximum 9.872).

Risk of bias was assessed using the Cochrane Collaboration’s Risk of Bias tool, and results are presented in a risk of bias graph (Supplementary Materials S2). In tumor-specific cancer studies (n = 16), most were judged as low risk for random sequence generation (13), allocation concealment (10), blinding (10), and incomplete outcome data (16), while all had some concerns for selective reporting and other bias. In studies of generic cancers (malignant only, n = 13), high risk of bias was frequently observed for blinding (7), selective reporting (13), and other bias (13), whereas most were low risk for randomization (10) and incomplete outcome data (12). For studies of generic cancers including both benign and malignant cases (n = 17), most were low risk across domains except for selective reporting, where all were rated as high risk.

## 4. Discussion

Opioids remain the cornerstone of postoperative pain management, as reflected in most RCTs, which typically include an opioid-based study arm. However, balancing the analgesic benefits of opioids with their adverse side effects presents ongoing challenges, such as nausea, constipation, dependence, and tolerance. Moreover, opioids necessitate intensive perioperative monitoring by a vigilant multidisciplinary team to prevent potential toxicity and respiratory depression. Additionally, concerns over opioid over-prescription, a major but often under-recognized risk factor for future misuse and addiction, have prompted increased scrutiny. Evidence suggests that escalating opioid doses may also contribute to chronic postoperative pain development [56,57]. The variability in analgesic outcomes across RCTs investigating opioid use could be attributed to differences in administration routes and opioid types.

To address the opioid-related side effects, non-opioid alternatives such as NSAIDs have gained prominence in postoperative pain management due to their effectiveness in reducing inflammation and alleviating pain. By inhibiting cyclooxygenase enzymes, NSAIDs decrease prostaglandin production, enhancing pain relief while minimizing opioid use. Studies have shown that NSAIDs can reduce postoperative pain compared to controls, with some demonstrating significant benefits such as Parecoxib [8,9,12,13,47]. However, results can vary, as other studies found no significant differences between certain NSAIDs, such as celecoxib and intravenous ketorolac [28,33,52]. Despite their benefits, NSAIDs can pose risks. These include gastrointestinal, renal, and cardiovascular adverse effects, particularly in patients with pre-existing conditions. Additionally, NSAIDs have been associated with increased anastomotic-related complications, such as fistulas and leaks, particularly in extensive cytoreductive procedures involving bowel resections and anastomoses [58]. A meta-analysis revealed that postoperative NSAID use increased the overall risk of anastomotic leakage, with non-selective NSAIDs (e.g., diclofenac) showing a higher risk. Conversely, selective NSAIDs and ketorolac were not significantly associated with this complication [59]. Therefore, careful selection of patients, medication, and monitoring are essential. When used appropriately, NSAIDs can significantly enhance multimodal analgesia strategies for patients undergoing gynecological cancer surgeries.

Other non-opioid options like paracetamol also play a crucial role in multimodal analgesia. Its analgesic effects are primarily due to the central inhibition of prostaglandin synthesis, making it an effective adjunct to opioid and NSAID therapies. One study found that metamizole (without ketoprofen) was inferior to paracetamol when combined with morphine on postoperative day 0. However, a multimodal approach incorporating morphine with either paracetamol and ketoprofen, or metamizole and ketoprofen provided equivalent analgesia, underscoring the effectiveness of paracetamol in postoperative pain management strategies [31].

As opioid-sparing strategies gain importance, agents like dexmedetomidine, an alpha-2 adrenergic agonist, have been increasingly utilized due to their sedative and analgesic properties. By inhibiting norepinephrine release, it induces sedation, reduced anxiety, and enhances pain relief. Studies have shown that dexmedetomidine infusion significantly reduces postoperative pain during activity at one h post-surgery but has no effect at rest or at other time points compared to control [27]. When compared to opioids, conflicting analgesic effects was observed with no significant difference between IV PCA dexmedetomidine and fentanyl [34]. Whereas combining dexmedetomidine and lidocaine significantly lowered pain levels and reduced opioid consumption, thereby minimizing the associated side effects compared to opioid alone [27,34,46]. Potential dexmedetomidine adverse effects include hypotension, bradycardia, and sedation-related complications, necessitating careful patient assessment and monitoring. When used judiciously, it can be a valuable adjunct in multimodal analgesia strategies.

Drug repurposing for postoperative pain management, leveraging the existing safety profiles of medications developed for other conditions, is an emerging strategy to accelerate the identification of effective, opioid-sparing analgesics. Sattari et al. investigated the use of duloxetine, a serotonin-noradrenaline reuptake inhibitor traditionally used for depression, anxiety, and chronic pain. Administering duloxetine two h prior to inducing analgesia significantly reduced pain scores in recovery and on the ward following abdominal hysterectomy for various gynecological indications compared to the control group [50]. This infers a possible alternative pathway in the management of postoperative pain. Anxiety and depression are well established factors influencing the severity of pain and vice versa. Duloxetine increases serotonin and norepinephrine levels which is associated with improved mental well-being whilst effectively managing neuropathic pain. Its dual action emphasizes the importance of integrating psychological well-being and neuropathic pain control for a comprehensive pain management, which is pertinent in major gynae-oncological surgery [60,61,62].

Furthermore, magnesium sulfate ought to be considered as an adjunct in pain management due to its antinociceptive effect. This occurs via the antagonization of NMDA receptors, preventing central sensitization and attenuates pre-existing hyperalgesia. Magnesium sulphate can be administered through various modalities (oral or parenteral) to alleviate pain and has been shown to be effective in cancer-related neurologic symptoms and chemotherapy-induced peripheral neuropathy [24]. While magnesium sulphate is increasingly recognized for its role in pain management, further research is required to elucidate its role as an adjuvant analgesic.

Regional anesthesia offers potential benefits as part of a multimodal approach to postoperative pain management, enhancing analgesic effectiveness and optimizing patient outcomes. Peripheral nerve blocks and fascial plane blocks, including ilioinguinal, iliohypogastric, and ESPB, have demonstrated effective pain relief [11,18], with ESPB outperforming TAPB by reducing tramadol use [20]. Magnesium-enhanced TAP blocks further improved analgesia [24], while intraperitoneal local anesthetics demonstrated limited benefits [22].

Central neuraxial approaches, including epidurals and spinal blocks, have shown effectiveness in certain studies, particularly for pain relief during ambulation and coughing [30,36]. However, overall efficacy is inconsistent when compared to peripheral nerve blockade, parenteral and oral options [25,29,43,44,49]. Complications such as hypotension, impaired mobilization and catheter-related complications may arise, necessitating intensive monitoring [30,36]. Variability in outcomes may be influenced by differences in the analgesics used. A tailored integration of anesthetic modalities could enhance pain management, facilitate earlier mobilization, and support recovery.

Postoperative cancer care involves pain management and addressing the psychological morbidity associated with cancer, which impacts both short- and long-term quality of life. Shi et al. demonstrated improved pain scores through standardized nursing care for cervical cancer patients, involving a dedicated quality control team trained in pain assessment, cancer pain psychology, and analgesic interventions, emphasizing medication compliance and the efficacy of pain medications [14,63]. Similarly, Zhu et al. showed that a standardized empathetic care model, incorporating training in empathetic care, communication, psychological support, interdisciplinary collaboration, enhanced postoperative recovery, improving pain, sleep, and psychological well-being, as reflected in a shorter hospital stay [18]. Future studies should explore integrating empathetic care into routine oncology nursing practice.

In addition to pharmacological approaches, complementary medicine has been considered for their potential to enhance pain relief. It remains a contentious area due to inconsistent standards in preparation and regulation leading to differing pharmacokinetics that ultimately impacts on patient’s response. Hou et al. examined the effects of Chinese herbal treatments combined with psychological care in patients with advanced cervical cancer, finding no significant difference in acute pain relief at day 1 and 7. However, combination therapy was more effective at minimizing pain on day 30 and 60 than Chinese herbal treatments alone [11].

Similarly, curcumin derived from turmeric has anti-inflammatory and antioxidant properties [64]. Ariyasriwatana et al. showed that the addition of curcumin significantly reduced the pain scores at 72 h, but not at 24 h following laparoscopic hysterectomy for multiple gynecology pathology including cancer. This may be attributed to its low bioavailability, with optimum dosage and method of administration yet to be established [37,65].

Acupuncture and massage therapy are alternative modalities that have been explored in pain management, though evidence for their efficacy post-gynecological surgery is limited. Lam et al. found no significant benefit of acupuncture on postoperative pain following laparotomy [45]. Taylor et al. compared Swedish massage with physiotone vibrational therapy on day 1 and 2 postoperatively. There was a non-significant trend towards improved pain scores following Swedish massage in comparison to usual care and physiotone vibrational therapy [32]. This is in keeping with a meta-analysis showing the benefits of massage therapy for surgery-related pain, with foot reflexology being more effective than body or aroma massage [66].

Abdominal binders may be considered in postoperative pain management to aid with mobilization, breathing, and coughing. Ossola et al. systemically reviewed the use of postoperative abdominal binding after midline laparotomy. They identified that abdominal binding significantly reduced postoperative pain on the first and third day and improved physical activity on the third day without impairment of respiratory function [67]. In keeping with this, Chantawong et al. also concluded that abdominal binders were effective at lowering pain scores on days 1 and 2, with no difference identified on day 3. In this trial, patients were not blinded to the intervention and this may have led to reporting bias [26].

Incorporating lifestyle measures such as exercise and diet further enhances the multimodal pain management approach. Early mobilization and structured exercise programs reduce pain, opioid use, and complications such as deep vein thrombosis [35]. It also promotes endorphin release, offering natural pain relief and enhancing functional recovery [68]. Additionally, diet plays a pivotal role in managing pain and recovery. Anti-inflammatory diet rich in fruits, vegetables, omega-3 fatty acids, and antioxidants helps reduce inflammation and pain. Adequate protein supports tissue healing, while stable blood glucose levels prevent delayed recovery. Hydration and electrolyte balance, particularly with magnesium, are also key in minimizing pain and enhancing healing. Incorporating exercise and a nutrient-rich diet into postoperative care can improve pain management and recovery, complementing traditional analgesic approaches [48,69].

Numerous studies have identified risk factors which may lead to suboptimal postoperative pain relief including: (1) age (increasing age may be associated with altered pain perception and metabolism of analgesics); (2) comorbidities (e.g., chronic pain conditions, previous surgery, psychological factors, high BMI); (3) medications (opioid tolerance, drug interactions); (4) lifestyle factors (substance misuse, smoking); (5) type and extent of surgery; (6) postoperative factors (inadequate or poor compliance pain management, complications); and (7) psychosocial factors (mental well-being, social support) [70,71]. These factors emphasize the complex interplay influencing pain, underscoring the requirements of a multidisciplinary approach and continuous assessment to optimize postoperative pain management.

Inadequate postoperative pain control can lead to prolonged bed rest and immobility, which increase the risk of complications such as venous thromboembolism, hospital-acquired infections, and functional decline. This issue is particularly pertinent in gynecologic oncology surgery, where extended hospitalization is common, especially among patients with vulvar cancer. The lack of specific studies addressing postoperative pain management in this subgroup highlights a significant gap in the literature. Effective pain control is imperative to mitigate immobility-related morbidity, reduce health economic burden, and improve overall outcomes [5,72].

Procedure-specific pain management is essential to optimize recovery and prevent chronic pelvic pain. While MIS patients are often presumed to exhibit less postoperative pain, this assumption may be influenced by variation in perioperative management standards. Patients undergoing radical open surgeries typically receive regional anesthesia and oversight from acute pain services, whereas MIS patients often do not [62,73]. Only 3 RCTs encompassed both MIS and open surgery, but neither compared postoperative pain outcomes amongst surgical approach.

The inclusion of both benign and malignant gynecologic surgeries introduces clinical variability that limits direct comparability. Furthermore, the aggregation of distinct gynecologic cancer subtypes presents additional complexity due to their differing biological behaviors and treatment paradigms. Future RCTs should implement stratification by surgical indication and cancer subtype to enhance the validity and applicability of findings.

## 5. Limitations

This review has several limitations that may affect the validity and generalizability of its findings. The included studies showed significant heterogeneity in design, sample size, and methodologies, complicating comparisons and precluded a quantitative meta-analysis. Variability in pain assessment tools and outcome measures hindered consistent data reporting. Most studies had short follow-up periods, limiting long-term evaluation of pain management and recovery. The lack of standardized pain management protocols and insufficient focus on multimodal approaches restricted insights into effective strategies. Many studies did not assess patient-reported outcomes or control for confounding variables, such as comorbidities and psychological factors, which are essential for understanding pain and recovery. Furthermore, the absence of subgroup or sensitivity analyses limits the interpretability of the review’s findings. Importantly, studies categorized as generic cancers and generic (benign and malignant) groups were at a higher risk of bias, largely due to selective reporting bias stemming from the absence of tumor-specific data breakdowns. This limitation may reduce confidence in the findings from these subsets and restrict the generalizability of the conclusions.

## 6. Conclusions

The limited cancer- and procedure-specific trials, combined with the diverse analgesic modalities employed, underscore the complexity of pain management, emphasizing the importance of a multimodal approach. Notably, no studies specifically addressed postoperative pain in vulval cancer, highlighting a significant gap in existing literature and underscoring the need for targeted research in this underrepresented patient group.

The studies revealed significant heterogeneity and a lack of standardized pain assessment methods. A consensus on quantifiable tools is essential for evaluating pain management efficacy. While no single analgesic modality proved superior, trends suggest multimodal approaches may offer better pain control. Additionally, integrating psychological well-being management is crucial alongside pharmacological relief.

These findings emphasize the urgent need for clear, standardized, and evidence-based guidelines to ensure equitable, effective, and individualized postoperative pain management across all gynecological cancer populations.

## Figures and Tables

**Table 1:** Summary of the included randomized control trials.

Paper, Author and Year	Country	Number of Patients	Type of Cancer	Postoperative Pain Monitoring Interval	Intervention	Validated Measurements
Cervical Cancer (n = 11)						
Effect of multimodal analgesia on gynecological cancer patients after radical resection Dong 2021 [8]	China	n = 98	Cervical (Laparoscopic radical resection)	Day 1ߝ3	Group 1: Multimodal analgesia Group 2: Control	NRS
Effect of flurbiprofen axetil on pain and cognitive dysfunction after radical operation of cervical cancer in elderly patients Dong 2022 [9]	China	n = 150	Cervical (Radical resection)	Not specified	Group 1: Control Group 2: Flurbiprofen axetil	VAS (0ߝ100)
Applied analysis of ultrasound-guided ilioinguinal and iliohypogastric nerve blocks in the radical surgery of aged cervical cancer Gu 2017 [10]	China	n = 62	Cervical (Radical resection) Stage Ia n = 23 Ib n = 27 IIa n = 12	Not specified	Group 1: US-guided ilioinguinal and iliohypogastric nerve blocks Group 2: Control	VAS
Effect of Psychological Care Combined with Traditional Chinese Medicine on Postoperative Psychological Stress Response in Patients with Advanced Cervical Cancer Hou 2021 [11]	China	n = 232	Cervical Stage IIIa n = 47; 48 IIIb n= 45; 47 IVa n = 24; 21 Histology SCC n = 92; 95 Adenocarcinoma n = 24; 21	Day 1ߝ60	Group 1: Chinese medicine treatment Group 2: Chinese medicine treatment and psychological care	VAS
A prospective, randomized, double-blind, placebo-controlled trial of acute postoperative pain treatment using opioid analgesics with intravenous ibuprofen after radical cervical cancer surgery Liu 2018 [12]	China	n = 59 (3 excluded)	Cervical (Radical surgery)	0ߝ48 h	Group 1: Placebo Group 2: Ibuprofen 400 mg Group 3: Ibuprofen 800 mg	VAS (0ߝ100)
Multi-dose parecoxib provides an immunoprotective effect by balancing T helper 1 (Th1), Th2, Th17 and regulatory T cytokines following laparoscopy in patients with cervical cancer Ma 2015 [13]	China	n = 80 (8 excluded)	Cervical (Laparoscopic radical hysterectomy) Stage Ib n = 14; 16 IIa n = 22; 20 Histology SCC n = 29; 32 Adenocarcinoma n = 6; 4 Adenosquamous n = 1; 0	0ߝ72 h	Group 1: Parecoxib Group 2: Control	VAS (0ߝ10)
Effect of quality control circle nursing mode on postoperative pain and anxiety of patients with cervical cancer Shi 2021 [14]	China	n = 324	Cervical Stage Ia n = 29; 32 Ib n = 15; 17 IIa n = 113; 106 IIb n = 5; 7 Histology SCC n = 138; 134 Adenocarcinoma n = 15; 18 Squamous adenocarcinoma n = 7; 9 Other n = 2; 1	0ߝ72 h	Group 1: Observation quality control circle nursing mode Group 2: Control routine nursing care	VAS (0ߝ10)
Use of various doses of S-ketamine in treatment of depression and pain in cervical carcinoma patients with mild/moderate depression after laparoscopic total hysterectomy Wang 2020 [15]	China	n = 417	Cervical (Laparoscopic modified radical hysterectomy)	Day 1ߝ7	Group 1: Control Group 2: Racemic Ketamine Group 3: High dose S Ketamine Group 4: Low dose S Ketamine	VAS
The efficiency of ultrasound-guided erector spinae plane block in early cervical cancer patients undergoing laparotomic radical hysterectomy: A double-blind randomized controlled trial Zhou 2023 [16]	China	n = 156 (2 excluded)	Cervical (Laparotomic radical hysterectomy)	2–24 h	Group 1: Erector spinae block (ESPB) Group 2: Transversus abdominus plane block (TAPB)	VAS (0–100)
Efficacy of oxycodone in intravenous patient-controlled analgesia with different infusion modes after laparoscopic radical surgery of cervical cancer a prospective, randomized, double-blind study Zhu 2019 [17]	China	n = 90 (7 excluded)	Cervical (Radical laparoscopic surgery)	0–38 h	Group 1: Oxycodone continuous infusion and bolus dose Group 2: Oxycodone bolus dose Group 3: PCA	VAS (0–10)
Impact of Perioperative Empathic Care on Postoperative Psychological Status in Patients with Cervical Cancer Zhu 2024 [18]	China	n = 196	Cervical Histology SCC n = 32; 31 Adenocarcinoma n = 32; 29 Adenosquamous n = 34; 38	Before treatment After nursing	Group 1: Conventional nursing Group 2: Empathetic care	NRS (0–10)
**Endometrial Cancer (n = 1)**						
Remifentanil injected during analepsia shortens length of postanesthesia care unit stay in patients undergoing laparoscopic surgery for endometrial cancer: a randomized controlled trial Zhu 2021 [19]	China	n = 99	Endometrial (Laparoscopic staging)	Before the patients were transferred back to the ward from postanesthesia care unit stay	Group 1: Bolus injection of Remifentanil Group 2: Bolus injection of Propofol	VAS (0–10)
**Ovarian Cancer (n = 4)**						
The use of erector spinae versus transversus abdominis blocks in ovarian surgery: A randomized, comparative study Abdullah 2022 [20]	Egypt	n = 60	Ovarian (Debulking)	0–24 h	Group 1: Ultrasound guided erector spinae block (ESPB) Group 2: Ultrasound guided transversus abdominis block (TAPB)	VAS
Patient-controlled thoracic epidural infusion with ropivacaine 0.375% provides comparable pain relief as bupivacaine 0.125% plus sufentanil after major abdominal gynecologic tumor surgery Gottschalk 2002 [21]	Germany	n = 30	Ovarian (Major abdominal gynecologic tumor surgery)	24–96 h	Group 1: Ropivacaine Group 2: Bupivacaine + Sufentanil Piritramide (breakthrough pain)	VAS (0–100mm)
Intraperitoneal ropivacaine reduces time interval to initiation of chemotherapy after surgery for advanced ovarian cancer: randomized controlled double-blind pilot study Hayden 2020 [22]	Sweden	n = 40	Ovarian (Laparotomy primary debulking surgery) Stage I n = 0; 1 II n = 0; 1 III n = 15; 11 IV n = 5; 7 Histology High grade serous n = 19; 15 Serous borderline n = 0; 1 Low grade serous n = 0; 2 Medium high grade serous n = 1; 1 Clear cell n = 0; 1	48 h	Group 1: Intraperitoneal Ropivacaine Group 2: Control	NRS (0–10)
Patient-controlled epidural analgesia reduces analgesic requirements compared to continuous epidural infusion after major abdominal surgery Standl 2003 [23]	Germany	n = 28	Ovarian (Debulking)	After 24 h of CEI on post operative day 1 and after 24 h of PCEA on post operative day 2	Group 1: CEI then PCEA with Ropivacaine Group 2: CEI then PCEA with Bupivacaine	VAS (0–100)
**Vulvar Cancer (n = 0)**						
**Generic Cancer (n = 13)**						
Efficacy of magnesium sulfate added to local anesthetic in a transversus abdominis plane block for analgesia following total abdominal hysterectomy: A randomized trial Abd-Elsalam 2017 [24]	Egypt	n = 60	Generic (Total abdominal hysterectomy) Ovarian Uterine	0–24 h	Group 1: TAPB with Bupivacaine + Magnesium sulfate Group 2: TAPB Bupivacaine	VAS (0–10)
Comparison of patient controlled epidural analgesia with continuous epidural analgesia for postoperative pain control after surgeries for gynecological cancers-a randomized controlled study Chandveettil 2021 [25]	India	n = 69 (9 excluded)	Generic (Midline laparotomy) Cervical n = 6 (radical hysterectomy) Ovarian n = 28 (cytoreductive surgery) Uterine n = 26 (staging surgery)	0–36 h	Group 1: CEA Group 2: PCEA	NRS (0–10)
Effect of Elastic Abdominal Binder on Pain and Functional Recovery Following Gynecologic Cancer Surgery: A Randomized Controlled Trial Chantawong 2021 [26]	Thailand	n = 120 (11 excluded)	Generic (Open major abdominal surgery) Cervical n = 34 Ovarian n = 35 Uterine n = 40	Day 1–3	Group 1: Abdominal binder Group 2: No binder	VAS
Effects of Perioperative Dexmedetomidine on Immunomodulation in Uterine Cancer Surgery: A Randomized, Controlled Trial Cho 2021 [27]	Republic of Korea	n = 100 (9 excluded)	Generic Cervical n = 25 Uterine n = 62 Myosarcoma n = 4	1–24 h	Group 1: Dexmedetomidine infusion Group 2: Control saline infusion	Numerical pain intensity scale (0–10)
Tenoxicam IV in major gynecological surgery—Pharmacokinetic, pain relief and hematological effects Jones 2000 [28]	Australia	n = 30	Generic (Laparotomy) Cervical Ovarian Uterine	0–48 h	Group 1: Control Group 2: Tenoxicam	VAS
Comparison of the analgesic effects continuous epidural anesthesia and continuous rectus sheath block in patients undergoing gynecological cancer surgery: a non-inferiority randomized control trial Kuniyoshi 2021 [29]	Japan	n = 100 (27 excluded)	Generic (Midline laparotomy) Cervical n = 35 Ovarian n = 10 Uterine n = 28	0–36 h	Group 1: CEA Group 2: CRSB	NRS
A comparison of patient controlled epidural analgesia with intravenous patient-controlled analgesia for postoperative pain management after major gynecologic oncologic surgeries: A randomized controlled clinical trial Moslemi 2015 [30]	Iran	n = 90	Generic (Major open gynecologic surgeries) Cervical n = 1 Endometrial n = 4 Ovarian n = 85	0–48 h First ambulation	Group 1: PCEA with Bupivacaine and Fentanyl Group 2: IV PCA with Fentanyl, Pethidine and Ondansetron	VAS (1–10)
Efficiency of postoperative pain management after gynecologic oncological surgeries with the use of morphine + acetaminophen + ketoprofen versus morphine + metamizol + ketoprofen Samulak 2011 [31]	Poland	n = 128	Generic (Laparotomy) Cervical Uterine Ovarian	Day 1–3	Group 1: Morphine SC, acetaminophen IV, Naproxen PR ± Ketoprofen IV Group 2: Morphine SC, Naproxen PR, Metamizole IV	NRS (0–10)
Effects of adjunctive Swedish massage and vibration therapy on short-term postoperative outcomes: A randomized, controlled trial Taylor 2003 [32]	USA	n = 146 (41 excluded)	Generic (Abdominal laparotomy) Generally ovarian masses	Day 0–3	Group 1: Massage Group 2: Physiotone Group 3: Usual care	Pain scale (0–10)
Adding ketoprofen to intravenous patient-controlled analgesia with tramadol after major gynecological cancer surgery: a double-blinded, randomized, placebo-controlled clinical trial Tuncer 2003 [33]	Turkey	n = 50	Generic Cervical n = 20 Uterine n = 10 Ovarian n = 20	0–24 h	Group 1: Control Group 2: Ketoprofen	VAS (1–10)
Effect of Dexmedetomidine Alone for Intravenous Patient-Controlled Analgesia After Gynecological Laparoscopic Surgery: A Consort-Prospective, Randomized, Controlled Trial Wang 2016 [34]	China	n = 40 (4 excluded)	Generic (Laparoscopic) Cervical Uterine	0–48 h	Group 1: Dexmedetomidine Group 2: Fentanyl	VAS (0–10)
Effects of preoperative walking on bowel function recovery for patients undergoing gynecological malignancy laparoscopy Xia 2022 [35]	China	n = 156	Generic (Laparoscopic) Cervical n = 53 Uterine n = 54 Ovarian n = 49	Day 1–3	Group 1: Routine usual care Group 2: Low and medium intensity walking exercise alongside routine nursing care	Prince Henry pain scoring standard (0–4)
The Effect of Perioperative Lidocaine Infusion on Postoperative Pain and Postsurgical Recovery Parameters in Gynecologic Cancer Surgery Yazici 2021 [36]	Turkey	n = 75	Generic (Wertheim and debulking surgery, pelvic and para-aortic lymph node dissection) Ovarian n = 34 Uterine n = 41	0–24 h	Group 1: Lidocaine—perioperative IV Lidocaine infusion Group 2: Opioid—PCA with Morphine Group 3: Epidural—PCA with Bupivacaine	VAS (0–10)
**Generic (Benign and Cancer) (n = 17)**						
Efficacy of Curcuminoids in Managing Postoperative Pain after Total Laparoscopic Hysterectomy: A Randomized Controlled, Open-Label Trial Ariyasriwatana 2022 [37]	Thailand	n = 98	Generic (Laparoscopic Hysterectomy) Cancer n = 5 (2; 3) Other n A = 93	24 and 72 h	Group 1: Curcumin capsules Group 2: Control—standard analgesia	VAS (10–point scale)
Oxycodone vs Sufentanil in Patient-Controlled Intravenous Analgesia After Gynecological Tumor Operation: A Randomized Double-Blind Clinical Trial Dang 2020 [38]	China	n = 140 Refusal of participating in the study n = 4 Excluded n = 12	Generic (Laparotomy or endoscopy)	3–48 h	Group 1: S = Sufentanil transition analgesia and Sufentanil PCIA Group 2: OS = Oxycodone transition analgesia and Sufentanil PCIA Group 3: SO = Sufentanil transition analgesia and Oxycodone PCIA Group 4: O = Oxycodone transition analgesia and Oxycodone PCIA	NRS (0–10)
Randomized controlled double-blind trial of transversus abdominis plane block versus trocar site infiltration in gynecologic laparoscopy ElHachem 2015 [39]	USA	n = 88	Generic (Laparoscopic) Cancer n = 32 (15; 17) Other n = 56	0–48 h	Group 1: Anesthesiologist-administered US guided TAPB Group 2: Laparoscopic guided TAPB Both groups, contralateral port sites were infiltrated with an equal amount of Bupivacaine in divided doses	VAS (0–10)
A prospective randomized trial comparing patient-controlled epidural analgesia to patient-controlled intravenous analgesia on postoperative pain control and recovery after major open gynecologic cancer surgery Ferguson 2009 [40]	USA	n = 153 randomized n = 135 evaluable	Generic (laparotomy) Cervical n = 3; 4 Uterine n = 17; 15 Ovarian n = 25; 26 Other n = 5; 4 Benign n = 36	Day 1–6	Group 1: PCEA Group 2: PCA	VAS (1–10)
Influence of General and Local Anesthesia on Postoperative Pain After a Loop Electrosurgical Excision Procedure Güngördük 2023 [41]	Turkey	n = 244	Generic (Loop electrosurgical excision procedure) Cervical n = 5; 9 Benign n = 118; 112	1–4 h	Group 1: Local anesthetic: Lidocaine spray applied to ectocervix, followed by 2 mL Bupivacaine submucosal injection Group 2: General anesthetic	Faces pain scale-revised and VAS (0–10)
The effects of fentanyl, oxycodone, and butorphanol on gastrointestinal function in patients undergoing laparoscopic hysterectomy: a prospective, double-blind, randomized controlled trial Guo 2022 [42]	China	n = 135 (23 excluded)	Generic (Laparoscopic hysterectomy) Cancer n = 51 (18; 15; 18) Other n = 84	0–48 h	Group 1: IV-PCA with Fentanyl Group 2: IV-PCA with Butorphanol Group 3: IV-PCA with Oxycodone	VAS (0–10)
The effects of Intrathecal morphine on patient-controlled analgesia, morphine consumption, postoperative pain and satisfaction scores in patients undergoing Gynecological Oncological surgery Kara 2012 [43]	Turkey	n = 60 (4 excluded)	Generic Cervical n = 3 Uterine n = 13 Ovarian n = 31 Other n = 9	0–48 h	Group 1: Intrathecal Morphine Group 2: Control	VAS (0–100mm)
Effect of intrathecal morphine and epidural analgesia on postoperative recovery after abdominal surgery for gynecologic malignancy: An open-label randomized trial Kjølhede2019 [44]	Sweden	n = 245 (168 excluded)	Generic (Radical midline laparotomy) Cervical n = 1; 0 Uterine n = 7; 13 Ovarian n = 13; 18 Other n = 1; 2 BOT n = 5; 0 Benign n = 17	Day 0–6.3	Group 1: EDA—standard regiment Group 2: ITM	NRS (0–10)
A combination of electroacupuncture and auricular acupuncture for postoperative pain after abdominal surgery for gynecological diseases: A randomized controlled trial Lam 2022 [45]	Hong Kong	n = 72	Generic (Laparotomy) Cervical n = 0 Uterine n = 6 Ovarian n = 19 Other n = 47	Day 0–5	Group 1: Acupuncture Group 2: Non-invasive sham acupuncture	NRS (0–11)
Efficacy of dexmedetomidine-based opioid-free anesthesia on the control of surgery-induced inflammatory response and outcomes in patients undergoing open abdominal hysterectomy Lotfy 2022 [46]	Egypt	n = 90	Generic (Open abdominal hysterectomy) Uterine n = 9 Other n = 81	0–24 h	Group 1: Opioid-based general anesthesia Group 2: Opioid-free general anesthesia Group 3: Epidural anesthesia	NRS (0–10)
Effects of parecoxib on morphine analgesia after gynecology tumor operation: A randomized trial of parecoxib used in postsurgical pain management Nong 2013 [47]	China	n = 80 (1 excluded)	Generic Cervical n = 21 Uterine n = 8 Ovarian n = 10 Other n = 40	2–48 h	Group 1: IV Parecoxib Group 2: Control	VAS (0–10)
Preoperative low-residue diet in gynecological surgery Palaia 2022 [48]	Italy	n = 168 (72 excluded)	Generic (Laparoscopic, laparotomy) Uterine n = 8; 5 Other n = 41; 42	12–24 h	Group 1: Low residue diet starting three days before surgery Group 2: Free diet	VAS
A randomized controlled trial of early oral analgesia in gynecologic oncology patients undergoing intra-abdominal surgery Pearl 2002 [49]	USA	n = 220	Generic (non-laparoscopic intra-abdominal surgery) Cervical n = 36 Uterine n = 53 Ovarian n = 70 Other n = 61	Day 0–2	Group 1: Oral Morphine Group 2: PCA parenteral Morphine, changed to scheduled oral Morphine on day 2 post op	Pain score (0–10)
Evaluating the effect of preoperative duloxetine administration on postoperative pain in patients under abdominal hysterectomy Sattari 2020 [50]	Iran	n = 60	Generic (Abdominal hysterectomy)	0–24 h	Group 1: Duloxetine Group 2: Control	VAS (0–10)
Does local infiltration anesthesia on laparoscopic surgical wounds reduce postoperative pain? Randomized control study Sugihara 2018 [51]	Japan	n = 322 (28 excluded)	Generic (Laparoscopic) Uterine (early stage) Other	1–2 h	Group 1: Local infiltration with Levobupivacaine Group 2: Control	VAS (0–10)
Celecoxib versus ketorolac following robotic hysterectomy for the management of postoperative pain: An open-label randomized control trial Ulm 2018 [52]	USA	n = 192 (54 excluded)	Generic (Open hysterectomy and robotic hysterectomy) Cervical and uterine n = 36	0–24 h	Group 1: Ketorolac IV Group 2: Preoperative oral Celecoxib followed by scheduled postoperative oral Celecoxib	VAS
Combination of Low-dose Nalbuphine and Morphine in Patient-controlled Analgesia Decreases Incidence of Opioid-related Side Effects Yeh 2009 [53]	Taiwan	n = 174 (5 excluded)	Generic (Total abdominal hysterectomy; myomectomy; ovarian tumor excision)	1–24 h	Group 1: Control PCA with Morphine Group 2: PCA with Morphine and Nalbuphine	NRS (0–10)

**Abbreviations**: CEA, continuous epidural analgesia; CEI, continuous epidural infusion; CRSB, continuous rectus sheath block; EDA, epidural analgesia; ESPB, erector spinae plane block; ITM, intrathecal morphine; IV, intravenous; PCA, patient-controlled analgesia; PCEA, patient-controlled epidural analgesia; PCIA, patient-controlled intravenous analgesia; PR, per rectum; SC, subcutaneous; TAPB, transversus abdominis plane block; US, ultrasound scan.

**Table 2:** Summary of pain scores categorized according to gynecological cancer type and pain score system used in each study.

	Author	Intervention	Study Size	Time Interval	*p* Value	Summary
Cervical cancer (n = 11)						
**NRS**	Dong 2021 [8]	Multimodal analgesia: 50 mg Flurbiprofen axetil IV before operation, 0.5% Ropivacaine LA infiltration, IV PCA post op (Flurbiprofen 100 mg, Sufentanil 100 µg, Morphine 2 mg in 100 mL normal saline BD, 1 mL bolus, lasting for 48 h)	n = 47	Day 1 Day 2 Day 3	**<0.05** **<0.05** **<0.05**	Pain scores decreased with the increase in time, and the scores of the multimodal analgesia group were lower than those of the conventional postoperative analgesia on the 1st, 2nd and 3rd days after operation.
		Conventional postoperative analgesia: IV PCA post op (Fentanyl 0.8 mg in 100 mL normal saline at 2 mL/h, 1 mL bolus)	n = 51			
	Zhu 2024 [18]	Conventional nursing	n = 98	Before and after treatment	**<0.001**	Perioperative empathetic care significantly improved postoperative pain (2.96 ± 0.84) compared with conventional nursing (4.36 ± 1.02).
		Empathetic care: empathetic nursing team, addressing psychological requirements, non-verbal and verbal compassionate communication, perioperative pain dynamically assessed and managed promptly, blood circulation check, protective measures for infection, small, frequent meals	n = 98			
**VAS**	Dong 2022 [9]	Flurbiprofen axetil 50 mg before anesthesia	n = 75	Not specified	**<0.05**	Clinical effect of Flurbiprofen axetil before anesthesia significantly improves pain control
		Control	n = 75			
	Gu 2017 [10]	IV Sufentanil + US-guided ilioinguinal + iliohypogastric nerve blocks (0.2 mL/kg ropivacaine)	n = 31	Not specified	**0.023**	US-guided ilioinguinal and iliohypogastric nerve blocks significantly improved the analgesic effects during the perioperative and postoperative period in cervical cancer radical surgeries.
		IV Sufentanil (induction) then Propofol + Sufentanil (maintenance)	n = 31			
	Hou 2021 [11]	Chinese herbal medicine treatment 4 courses of 15 days of a combination of Chinese herbal treatments (Chinese medicine treatment = galanga galangal fruit 10 g, white mulberry root bark 10 g, heartleaf houttuynia herb 30 g, eucommia bark 20 g, poria 10 g, milkvetch root 30 g, large head atractylodes rhizome 15 g, coix seed 20 g, garden burnet root 25 g, willow leaf rhizome 5 g, Chinese angelica 5 g, cassia bark 30 g, light yellow sophora root 15 g, peony root 10 g, milkwort root 15 g, and adactylies rhizome 25 g)	n = 116	Day 1 Day 7 Day 30 Day 60	0.852 0.556 **0.002** **≤0.001**	Difference in pain scores between the two groups was not statistically significant on day 1 or 7 of treatment. At day 30 and 60 of treatment, the difference in pain score between the two groups was statistically significant. Psychological care combined with traditional Chinese medicine in the treatment of advanced cervical cancer patients after surgery was effective reducing pain level.
		Chinese medicine treatment (as above) and psychological care (effective communication, health education, understand patient’s condition and needs, encourage patient to talk about concerns, encourage family involvement, music therapy)	n = 116			
	Liu 2018 [12]	Placebo (rest)	n = 20	1 h 3 h 6 h 12 h 24 h 36 h 48 h	**0.049** >0.05 >0.05 >0.05 >0.05 >0.05 >0.05	Ibuprofen 800 mg was associated with a significant reduction in pain intensity at rest at 1 h, and with movement at 24 h post administration. Pain intensity at rest or with movement at other time points was not significantly different between the three groups.
		Ibuprofen 400 mg every 6 h for 48 h (rest)	n = 17			
		Ibuprofen 800 mg every 6 h for 48 h (rest)	n = 19			
		Placebo (movement)	n = 20	1 h 3 h 6 h 12 h 24 h 36 h 48 h	>0.05 >0.05 >0.05 >0.05 **0.04** >0.05 >0.05	
		Ibuprofen 400 mg every 6 h for 48 h (movement)	n = 17			
		Ibuprofen 800 mg every 6 h for 48 h (movement)	n = 19			
	Ma 2015 [13]	Parecoxib 40 mg prior to surgery and 12 hly post surgery until the 60 h time point (rest)	n = 36	Basal 2 h 6 h 12 h 18 h 24 h 36 h 48 h 60 h 72 h	>0.05 **<0.05** **<0.05** **<0.05** >0.05 >0.05 >0.05 >0.05 >0.05 >0.05	Pain scores at rest for Parecoxib group were significantly reduced at 2, 6 and 12 h post-surgery. After movement, patients in the Parecoxib group experienced reduced pain at 2, 6, 12, 18 and 24 h post-surgery. Parecoxib appears to exert a stronger analgesic effect following laparoscopy.
		Control normal saline at the same time points (rest)	n=36			
		Parecoxib 40 mg prior to surgery and 12 hly post surgery until the 60 h time point (movement)	n = 36	Basal 2 h 6 h 12 h 18 h 24hr 36 h 48 h 60 h 72 h	>0.05 **<0.05** **<0.05** **<0.05** **<0.05** **<0.05** >0.05 >0.05 >0.05 >0.05	
		Control—normal saline at the same time points (movement)	n = 36			
	Shi 2021 [14]	Quality control circle nursing mode: Nursing with a focus on improving postoperative pain	n = 162	6 h 24 h 48 h 72 h	**<0.001** **<0.001** **<0.001** **<0.001**	Pain scores at 6 h, 24 h, 48 h and 72 h in the quality control circle nursing group were lower than those in the routine nursing care group.
		Routine nursing care	n = 162			
	Wang 2020 [15]	Control: 50 mL normal saline IV after 1 h of analgesia	n = 105	Day 1 Day 2 Day 3 Day 5 Day 7	**<0.05** in all treatment groups at days 1, 2 and 3	In all treatment groups, the pain scores at 1, 2, and 3 days were remarkably lower than in the saline group (*p* < 0.05). The high dose S-Ketamine (0.5 mg/kg) group showed the lowest pain scores, but no significant difference was observed between the low-dose S-Ketamine (0.25 mg/kg) group and the racemic Ketamine group. After 5 and 7 days, the VAS scores were reduced to the baseline in all groups. Results indicate that S-ketamine had better efficacy in reducing short-term postoperative pain than the same dose of racemic Ketamine
		Racemic Ketamine 50 mL 0.5 mg/kg IV after 1 h of analgesia	n = 104			
		High dose S-Ketamine 50 mL 0.5 mg/kg IV after 1 h of analgesia	n = 104			
		Low dose S-Ketamine 5 mL 0.25 mL/kg IV after 1 h of analgesia	n = 104			
	Zhou 2023 [16]	Erector spinae block (ESPB) with 20 mL injection of 0.375% Ropivacaine bilaterally + PCIA (rest)	n = 77	2 h 4 h 6 h 12 h 24 h	**0.003** **<0.001** **<0.001** **<0.001** **0.012**	There was less analgesic consumption and Sufentanil consumption in the PCIA pump in the ESPB group. The pain scores at rest were significantly lower in this group at all time points up to 12 h at both rest and on coughing. The ESPB group required fewer rescue analgesia and higher analgesia satisfaction.
		Transversus abdominus plane block (TAPB) with 20 mL injection of 0.375% Ropivacaine bilaterally + PCIA (rest)	n = 77			
		Erector spinae block (ESPB) with 20 mL injection of 0.375% Ropivacaine bilaterally + PCIA (cough)	n = 77	2 h 4 h 6 h 12 h 24 h	**0.003** **<0.001** **0.004** **<0.001** 0.112	
		Transversus abdominus plane block (TAPB) with 20 mL injection of 0.375% Ropivacaine bilaterally + PCIA (cough)	n = 77			
	Zhu 2019 [17]	Oxycodone with continuous infusion of 0.01 mg/kg/h and 0.03 mg/kg bolus dose (rest)	n = 27	1 h 6 h 12 h 24 h 48 h	**0.003** **0.007** 0.083 0.051 **0.006**	There are significant differences in the pain scores when resting or coughing among the 3 groups at 1, 6, and 48 h postoperatively. Oxycodone with 0.03mg/kg bolus dose group had a higher pain score than the other 2 groups at 1, 6, and 48 h. There were no differences between Oxycodone with continuous infusion of 0.01 mg/kg/h and 0.03 mg/kg bolus dose group.
		Oxycodone with 0.03 mg/kg bolus dose (rest)	n = 27			
		PCA administered as a time-scheduled decremental continuous infusion based on lean body mass (rest)	n = 29			
		Oxycodone with continuous infusion of 0.01 mg/kg/h and 0.03 mg/kg bolus dose (cough)	n = 27	1 h 6 h 12 h 24 h 48 h	**0.033** **0.006** 0.150 0.111 **0.002**	
		Oxycodone with 0.03 mg/kg bolus dose (cough)	n = 27			
		PCA administered as a time-scheduled decremental continuous infusion based on lean body mass (cough)	n = 29			
**Endometrial Cancer (n = 1)**						
**VAS**	Zhu 2021 [19]	Remifentanil 1 μg/kg prior to extubation if patient moved unconsciously	n = 51	Before the patients were transferred back to the ward from post anesthesia care unit stay	0.82	Pain score was comparable between the two groups
		Propofol 1 mg/kg prior to extubation if patient moved unconsciously	n = 48			
**Ovarian Cancer (n = 4)**						
**NRS**	Hayden 2020 [22]	Intraperitoneal Ropivacaine after opening peritoneal cavity 40 mL Ropivacaine 1mg/mL instilled to coat the peritoneum. This was repeated at 4 h and at the end of surgery. Prior to closing, a catheter was inserted into the pelvis and connected to an infusion pump (10 mL Ropivacaine 2 mg/mL every other h for 72 h)	n = 20	48 h	0.053	Pain intensity was similar in the two groups.
		Placebo—Ropivacaine was replaced with normal saline at the same time points	n = 20			
**VAS**	Abdullah 2022 [20]	Ultrasound guided ESPB with 20 mL 0.25% Bupivacaine bilaterally	n = 30	0–30 min 2–4 h 6 h 12 h 18 h 24 h	**<0.006** **<0.003** **<0.001** 0.3 **<0.001** 0.05	VAS scores were significantly lower in the ESPB group with the exception of time points 12 h and 24 h (almost significant, *p* = 0.05). There was a longer time to first analgesic request in the ESPB group. All patients in the TAPB group required rescue Tramadol compared to just 60% of the ESPB group.
		Ultrasound guided TAPB with 20 mL 0.25% Bupivacaine bilaterally	n = 30			
	Gottschalk 2002 [21]	PCEA with 10 mL Ropivacaine 0.375% (cough)	n = 30 (does not specify number per group)	24 h 36 h 48 h 60 h 72 h 84 h 96 h	>0.05 >0.05 >0.05 >0.05 >0.05 >0.05 >0.05	No significant differences in pain scores between the two groups
		PCEA with 10 mL Bupivacaine 0.125% + Sufentanil 0.5 µg/mL (cough)				
		PCEA with 10 mL Ropivacaine 0.375% (mobilization)	n = 30 (does not specify number per group)	24 h 36 h 48 h 60 h 72 h 84 h 96 h	0.9 >0.05 0.93 >0.05 0.78 >0.05 0.49	
		PCEA with 10 mL Bupivacaine 0.125% + sufentanil 0.5 µg/mL (mobilization)				
	Standl 2003 [23]	24 h CEI (6–10 mL/h 0.2% Ropivacaine) followed by PCEA (Ropivacaine 0.2% every 20 min) (rest)	n = 14	Day 1 Day 2	>0.05 >0.05	There were no differences in the pain scores at rest. The authors state that on coughing and during mobilization out of bed, patients in the bupivacaine group showed lower pain scores on day 1. Also, patients in the ropivacaine group had higher pain scores during CEI when compared with the following 24 h using PCEA.
		24 h CEI (6–10 mL/h 0.125% Bupivacaine and 0.5 µg/mL Sufentanil) followed by PCEA (Bupivacaine 0.125% and 0.5 µg/mL Sufentanil every 20 min) (rest)	n = 14			
		24 h CEI (6–10 mL/h 0.2% Ropivacaine) followed by PCEA (Ropivacaine 0.2% every 20 min) (coughing)	n = 14	Day 1 Day 2	No *p* values	
		24 h CEI (6–10 mL/h 0.125% Bupivacaine and 0.5 µg/mL Sufentanil) followed by PCEA (Bupivacaine 0.125% and 0.5 µg/mL Sufentanil every 20 min) (coughing)	n = 14			
		24 h CEI (6–10 mL/h 0.2% Ropivacaine) followed by PCEA (Ropivacaine 0.2% every 20 min) (mobilization)	n = 14	Day 1 Day 2	No *p* values	
		24 h CEI (6–10 mL/h 0.125% Bupivacaine and 0.5 µg/mL Sufentanil) followed by PCEA (Bupivacaine 0.125% and 0.5 µg/mL Sufentanil every 20 min) (mobilization)	n = 14			
**Generic Cancer (n = 13)**						
**Numerical pain intensity scale (0–10)**	Cho 2021 [27]	Dexmedetomidine infused at rates of 0.4 mg/kg/h intraoperatively and 0.15 mg/kg/h during the first 24 h postoperatively (at rest)	n = 46	1 h 24 h 48 h	0.339 0.888 0.493	Pain severity with activity during the first 1 h was significantly less in the Dexmedetomidine group. The authors cite that Dexmedetomidine reduced postoperative pain in the early post operative period, however this was not statistically significant at rest or at 24 h and 48 h with activity.
		Control saline infused at the same rate (Rest)	n = 45			
		Dexmedetomidine infused at rates of 0.4 mg/kg/h intraoperatively and 0.15 mg/kg/h during the first 24 h postoperatively (Activity)	n = 46	1 h 24 h 48 h	**0.016** 0.629 0.553	
		Control saline infused at the same rate (Activity)	n = 45			
**NRS (0–10)**	Chandveettil 2021 [25]	PCEA, background epidural infusion of 0.1% Ropivacaine with Fentanyl 2 mcg/mL at 6 mL/h after bolus of 6ml postoperatively. Bolus of 4 mL with a lock out interval of 30 min	n = 30	0–6 h 0–24 h 0–36 h	0.381 0.676 0.896	There is no significant difference. Postoperative pain relief with CEI and PCEI is comparable
		CEA, background epidural infusion of 0.1% Ropivacaine with Fentanyl 2 mcg/mL at 6 mL/h after bolus of 6 mL postoperatively. Rate increased to 10 mL/h by staff depending on pain score	n = 30			
	Kuniyoshi 2021 [29]	CEA, Levobupivacaine 0.25% 10 mL = 25 mg before and just after surgery, continued at 5 mg/h	n = 37	4 h 6 h 8 h 12 h 24 h 36 h	No *p* values specified	CRSB is significantly superior to CEA at rest at 24 h postsurgery only. No significant difference at any other time point at rest or movement.
		CRSB, 0.2%, 20 mL = 40 mg Levobupivacaine administered on each side before and just after surgery, continued at 7.5 mh/h on each side	n = 36			
	Samulak 2011 [31]	Morphine 1 mg/kg SC 4 hly, 1 g acetaminophen IV 6 hly, 500 mg naproxen PR	n = 64	Day 0 Day 1 Day 2 Day 3 Day 4 or >	**<0.05** No other *p* values	The combination of morphine, acetaminophen and ketoprofen or morphine, Metamizole and ketoprofen gives satisfactory pain relief. Metamizole provided less pain relief than acetaminophen on the day of surgery.
		Morphine 1 mg/kg SC 4 hly, 1 g Metamizole IV 6 hly, 500 mg Naproxen PR	n = 64			
**Pain scale (0–10)**	Taylor 2003 [32]	45-min massage after surgery and on days 1 and 2 postoperative	n = 34	Day 0 Day 1 Day 2	No *p* values	After controlling for multiple comparisons and outcomes, no significant differences were demonstrated between the groups, though there was a trend in favor of massage therapy
		Physiotone vibrational medicine–20 min session on the evening after surgery and on days 1 and 2 post operative	n = 35			
		Usual care	n = 36			
**Prince Henry pain scoring standard**	Xia 2022 [35]	Low and medium intensity walking exercise for at least 150 min a week before operation, alongside routine nursing care	n = 78	24 h 48 h 72 h	0.801 **<0.001** **<0.001**	Beyond 24 h, there is a significant improvement in abdominal pain scores in the group who underwent the walking exercise program.
		Routine nursing care	n = 78			
**VAS**	Abd-Elsalam 2017 [24]	US guided TAPB with 20 mL 0.25% Bupivacaine and 2 mL Magnesium Sulfate 10% on each side of abdominal wall (intervention)	n = 30	1 h 2 h 4 h 6 h 8 h 10 h 12 h	**<0.05** **<0.05** **<0.05** **<0.05** >0.05 **<0.05** **<0.05**	The mean postoperative pain score was significantly lower in the intervention group at all time points until after 8 h, where there was an insignificant difference between both groups. At 10 and 12 h, there was a significantly lower VAS score in the control group. At 24 h, the VAS scores were significantly lower in the intervention group.
		US guided TAPB with 20 mL 0.25% Bupivacaine on each side of abdominal wall (control)	n = 30			
	Chantawong 2021 [26]	Abdominal binder on days 1–7	n = 56	Baseline Day 1 Day 2 Day 3	**0.02** **0.01** **0.03** 0.13	The baseline, postoperative day 1 and 2 pain scores for the intervention group were significantly lower. There was no significant difference in the postoperative day 3 pain score or in the change in postoperative day 1–3 pain scores from the baseline.
		Control	n = 53			
	Jones 2000 [28]	IV Tenoxicam pre surgery (20mg diluted in 2 mL)	n = 15	4 h 24 h 48 h	0.08 0.793 0.422	There was no significant difference between the two groups at rest and on leg raising.
		Placebo pre surgery (2 mL normal saline with vitamin B complex as coloring)	n = 15			
	Moslemi 2015 [30]	PCEA, Bupivacaine 0.5% 120 mg and Fentanyl 150 µg in normal saline (100 mL) at rate of 6–8 mL/h with bolus of 2 mL every 15 min as needed	n = 45	2 h 4 h 8 h 12 h 24 h 48 h Mean	No significant difference at any time point, *p* > 0.05 **<0.001**	There was no significant difference in pain score at any of the time points. The mean severity of pain at first ambulation was significantly lower in the PCEA group (*p* < 0.001)
		IV PCA, 300 µg (6 mL) Fentanyl, 200 mg (4 mL) Pethidine in normal saline (100 mL). Infusion initially set to 6–8 mL/h with bolus of 2 mL every 15 min as needed	n = 45			
	Tuncer 2003 [33]	IV Ketoprofen 100 mg bolus at the end of surgery	n = 25	6 h 12 h 18 h 24 h	No significant difference in pain scores, *p* > 0.05	There was no significant difference in pain scores between the two groups. The cumulative PCA-Tramadol consumption was significantly lower in Ketoprofen treated patients (*p* < 0.05)
		Placebo (IV normal saline at the end of surgery)	n = 25			
	Wang 2016 [34]	Dexamedetomidine IV PCA 0.25 µg/kg/h dminiluted to 100 mL–2 mL/h infusion with 1ml bolus dose with 15 min lockout	n = 20	4 h 6 h 8 h 24 h 48 h	0.120 0.594 0.835 0.451 0.881	There was no significant difference between groups.
		Fentanyl IV PCA 20 µg/kg/h diluted to 100 mL – 2 mL/h infusion with 1 mL bolus dose with 15 min lockout	n = 16			
	Yazici 2021 [36]	Lidocaine: intraoperative and postoperative IV Lidocaine infusion (1.5 mg/kg bolus at induction, 1.5 mg/kg/h until 24 h postop) and PCA with IV Morphine (1 mg bolus with 10 min lockout) (rest)	n = 25	15 min 30 min 60 min 2 h 6 h 12 h 24 h 24 h	0.51 0.23 0.19 0.17 0.50 0.07 **0.01** Epidural vs. Opioid: **0.003**	Pain scores (rest) at 24 h and pain scores (cough) at 12 and 24 h were significantly lower in the epidural group than in the opioid group. VAS scores were found to be similar between the Lidocaine and epidural group
		Opioid: intraoperative IV saline infusion, Remifentanil infusion and postoperative PCA with IV Morphine (1 mg bolus with 10 min lockout) (rest)	n = 25			
		Epidural: intraoperative IV saline infusion, Remifentanil infusion and postoperative PCA with epidural Bupivacaine (4 mL 0.125% bolus with 20 min lockout) (rest)	n = 25			
		Lidocaine: intraoperative and postoperative IV Lidocaine infusion (1.5 mg/kg bolus at induction, 1.5 mg/kg/h until 24 h postop) and PCA with IV Morphine (1mg bolus with 10 min lockout) (cough)	n = 25	15 min 30 min 60 min 2 h 6 h 12 h 24 h 12 h 24 h	0.15 0.39 0.16 0.15 0.05 **0.02** **0.02** Epidural vs. Opioid: **0.004** **0.004**	
		Opioid: intraoperative IV saline infusion, Remifentanil infusion and postoperative PCA with IV Morphine (1 mg bolus with 10 min lockout) (cough)	n = 25			
		Epidural: intraoperative IV saline infusion, Remifentanil infusion and postoperative PCA with epidural Bupivacaine (4 mL 0.125% bolus with 20 min lockout) (cough)	n = 25			
**Generic (Benign and Cancer) (n = 17)**						
**NRS**	Dang 2020 [38]	Sufentanil (0.1 µg/kg for laparoscopy or 0.15 µg/kg for laparotomy) transition analgesia followed by Sufentanil PCIA for 48 h (2 µg bolus with a 5 min lockout and background infusion of 2 m/h)	n = 32	3 h 24 h 48 h	**<0.0083**	Both Sufentanil and Oxycodone PCIA provided adequate pain relief in transitional analgesia and PCIA treatment. Patients who received the oxycodone transition analgesia had lower pain scores at rest and coughing. Oxycodone showed less analgesic drug consumption and faster recovery than Sufentanil.
		Oxycodone (0.1 mg/kg laparoscopy or 0.15 mg/kg for laparotomy) transition analgesia and Sufentanil PCIA for 48 h (2 µg bolus with a 5 min lockout and background infusion of 2 m/h)	n = 30			
		Sufentanil (0.1 µg/kg for laparoscopy or 0.15 µg/kg for laparotomy) transition analgesia and Oxycodone PCIA for 48 h (2 mg bolus’ with a 5 min lockout, no background infusion)	n = 30			
		Oxycodone (0.1 mg/kg laparoscopy or 0.15 mg/kg for laparotomy) transition analgesia and Oxycodone PCIA for 48 h (2 mg bolus’ with a 5 min lockout, no background infusion)	n = 32			
	Kjølhede 2019 [44]	Epidural analgesia with bolus of Fentanyl 50–100 µg and a bolus of Bupivacaine 2.4 mg/mL, adrenaline 2.4 µg/mL, fentanyl 1.8 µg/mL, which was continued as an infusion (at rest)	n = 39	Days 0–6.3	0.34	There was no significant difference in the overall assessment of pain between the two groups
		Intrathecal combined Bupivacaine 15 mg, Morphine 0.2 mg and Clonidine 75 µg (at rest)	n = 38			
		Epidural analgesia with bolus of Fentanyl 50–100 µg and a bolus of Bupivacaine 2.4 mg/mL, Adrenaline 2.4 µg/mL, Fentanyl 1.8 µg/mL, which was continued as an infusion (mobilization)	n = 39	Days 0–6.3	0.08	
		Intrathecal combined Bupivacaine 15 mg, Morphine 0.2 mg and Clonidine 75 µg (mobilization)	n = 38			
	Lam 2022 [45]	Acupuncture 2 h prior to surgery, immediately upon arrival to ward and then daily during hospital stay up to postoperative day 5 (rest)	n = 36	Days 0–5	0.439	Perioperative acupuncture was to be superior to sham acupuncture in controlling post laparotomy pain
		Sham acupuncture 2 h prior to surgery, immediately upon arrival to ward and then daily during hospital stay up to postoperative day 5 (rest)	n = 36			
		Acupuncture 2 h prior to surgery, immediately upon arrival to ward and then daily during hospital stay up to postoperative day 5 (cough)	n = 36	Days 0–5	0.727	
		Sham acupuncture 2 h prior to surgery, immediately upon arrival to ward and then daily during hospital stay up to postoperative day 5 (cough)	n = 36			
	Lotfy 2022 [46]	Opioid based GA (Fentanyl 1 µg/kg)	n = 30	24 h	**<0.001**	Pain scores were significantly lower in the opioid free group.
		Opioid free GA (loading dose of 0.6 µg/kg D and 1.5 mg/kg Lidocaine, followed by infusion)	n = 30			
		Epidural anesthesia (loading dose of 15 mL 0.5% Bupivacaine and intermittent doses as required)	n = 30			
	Yeh 2009 [53]	100 mg Morphine and 1 mg Nalbuphine in 100 mL normal saline PCA	n = 83	1 h 2 h 4 h 24 h	No *p* values	The pain scores did not differ significantly between the two groups throughout the observation period
		100 mg Morphine in 100 mL normal saline PCA	n = 86			
**Pain score (0–10)**	Pearl 2002 [49]	Early oral analgesia oral Morphine on day 1: scheduled dose of non-sustained release oral Morphine 20mg every 4 h, with additional 10mg every 2 h as needed	n = 113	Day 0 Day 1 Day 2	No significant difference in pain scores	There was no significant difference between the pain scores in the two groups.
		Parenteral analgesia Day 1 PCA parenteral Morphine continued then on day 2 scheduled oral and basal parenteral doses were discontinued	n = 107			
**VAS**	Ariyasriwatana 2022 [37]	Standard analgesia + Curcumin capsules (Curcuminoid curcumin, Demethoxycurcumin, and Bisdemethoxycurcumin with 20 mL of Turmeric oils, e.g., Tumerone, Atlantone, and Zingiberone) 100 mg QDS	n = 49	24 h 72 h	0.129 **0.001**	There was a significant difference in the pain scores in favor of the use of curcumin capsules at 72 h, but not at 24 h.
		Standard analgesia (control)	n = 49			
	El Hachem 2015 [39]	US guided TAPB with 30 mL 0.25% Bupivacaine with Epinephrine	n = 45	1 h 2 h 4 h 6 h 8 h 12 h 18 h 24 h 48 h Overall	0.274 **0.004** 0.064 0.071 0.137 0.228 0.256 0.412 0.780 **0.001**	Although TAP blocks achieved postoperative pain scores that are comparable with high volume local port side infiltration, there was only a significant difference in favor of the TAP blocks in the US guided group only.
		Patients served as their own control, local infiltration of 30 mL 0.25% Bupivacaine with Epinephrine in divided doses into contralateral side				
		Laparoscopic guided TAPB with 30 mL 0.25% Bupivacaine with Epinephrine	n = 43	1 h 2 h 4 h 6 h 8 h 12 h 18 h 24 h 48 h Overall	0.323 0.613 0.415 0.164 0.350 0.295 0.560 0.561 0.736 0.352	
		Patients served as their own control, local infiltration of 30 mL 0.25% Bupivacaine with Epinephrine in divided doses into contralateral side				
	Ferguson 2009 [40]	IV morphine PCA continuous basal rate 1 mg/h with rescue bolus of 1mg every 10 min (at rest)	n = 68	Day 1 Day 1–3 Day 6	**0.01** **<0.05** **0.028**	There was a significant difference in the pain scores between the two groups, both at rest and on coughing. Patients with PCEA had significantly less pain.
		PCEA with Morphine 100 µg/mL and Bupivacaine 0.05% at continuous basal rate of 4 mL/h with rescue bolus of 4 mL every 30 min as needed (at rest)	n = 67			
		IV morphine PCA continuous basal rate 1 mg/h with rescue bolus of 1 mg every 10 min (cough)	n = 68	Day 1 Day 1–3 Day 6	**<0.03** **<0.03** **0.003**	
		PCEA with Morphine 100 µg/mL and Bupivacaine 0.05% at continuous basal rate of 4 mL/h with rescue bolus of 4 mL every 30 min as needed (cough)	n = 67			
	Güngördük 2023 [41]	Local anesthetic: 50 mg Lidocaine spray was applied to ectocervix, followed by 2 mL Bupivacaine submucosal injection	123	1 h 2 h 4 h 2 weeks	0.118 0.052 0.206	Although the mean VAS scores were higher in the LA group, the difference was not significant
		General anesthetic	121			
	Guo 2022 [42]	Fentanyl IV PCA 8.3 µg/kg in 100 mL normal saline with a background infusion rate of 2 mL/h, bolus dose of 3 mL with 15 min lockout and infusion time of 48 h	n = 39	4 h 12 h 24 h 48 h	Overall *p* value = 0.517	There was no difference in pain scores amongst the three groups
		Oxycodone IV PCA 0.5 mg/kg in 100 mL normal saline with a background infusion rate of 2 mL/h, bolus dose of 3 mL with 15 min lockout and infusion time of 48 h	n = 36			
		Butorphanol IV PCA 0.16 mg/kg in 100 mL normal saline with a background infusion rate of 2 mL/h, bolus dose of 3 mL with 15 min lockout and infusion time of 48 h	n = 37			
	Kara 2012 [43]	0.3 mg ITM and PCA (Morphine bolus of 0.05 mg/kg)	n = 28	30 min 1 h 3 h 6 h 12 h 24 h 48 h	No *p* values	No significant difference between the groups at all time points. The study showed that ITM significantly reduced the cumulative Morphine consumption without causing a significant difference in pain and satisfaction scores or the rate of side effects.
		PCA (Morphine bolus of 0.05 mg/kg)	n = 28			
	Nong 2013 [47]	IV Parecoxib 40 mg (2 mL) 20 min before induction of anesthesia, followed by 40 mg every 12 h for 48 h after the operation (at rest)	n = 39	2 h 6 h 12 h 24 h 48 h	**<0.05** **<0.05** **<0.05** **<0.05** **<0.05**	Pain scores at rest and on movement in the Parecoxib group were significantly lower than the control group at all time points. Parecoxib administered with Morphine provided greater pain relief than morphine alone.
		Control—IV Saline at the same time points (at rest)	n = 40			
		Study—IV Parecoxib 40 mg (2 mL) 20 min before induction of anesthesia, followed by 40 mg every 12 h for 48 h after the operation (movement)	n = 39	2 h 6 h 12 h 24 h 48 h	**<0.05** **<0.05** **<0.05** **<0.05** **<0.05**	
		Control IV Saline at the same time points (movement)	n = 40			
	Palaia 2022 [48]	Low residue diet starting three days before surgery	n = 49	12 h 24 h 48 h	0.348 0.309 0.0502	There was no difference in pain between the two groups. Analgesic request was marginally lower (though was not significant) in the low residue diet group (4.1% v 17.1%; OR 0.21 (95% CI, 0.04–1.03); *p* = 0.06).
		Free diet	n = 47			
	Sattari 2020 [50]	Duloxetine capsules 60 mg 2 h prior to analgesia	n = 30	In recovery On ward	**0.006** **0.001**	Those who had Duloxetine experienced significantly less pain.
		Control Starch capsules	n = 30			
	Sugihara 2018 [51]	Local infiltration with 2 mL 0.5% levobupivacaine per 1 cm of wound into muscle fascia at the end of laparoscopic surgery	n = 147	1 h 2 h	0.34 0.28	The pain scores were not significantly different between the two groups overall. However, at 2 h post op, there was a significant difference between the two groups in those who had undergone a laparoscopic assisted vaginal hysterectomy (*p* = 0.047) and laparoscopic hysterectomy (*p* = 0.007).
		Control with saline infiltration	n = 147			
	Ulm 2018 [52]	400 mg Celecoxib PO 1 h prior to surgery, 200 mg Celecoxib PO BD post op to complete 7 days	n = 68	Average inpatient score	0.21	There were no differences in inpatient pain scores in the immediate postoperative period.
		Ketorolac IV 30 mg 6 hly for 48 h post op or until discharge	n = 70			

Bold values indicate statistically significant results (*p* < 0.05). Abbreviation: CEA, continuous epidural analgesia; CEI, continuous epidural infusion; CRSB, continuous rectus sheath block; ESPB, erector spinae plane block; GA, general anesthesia; ITM, intrathecal morphine; IV, intravenous; LA, local anesthesia; NRS, numerical rating scale; PCA, patient-controlled analgesia; PCEA, patient-controlled epidural analgesia; PCIA, patient-controlled intravenous analgesia; TAPB, transversus abdominis plane block; US, ultrasound scan; VAS, Visual Analog Scale.

**Table 3:** Summary of the type of analgesia and mode of delivery in each study.

	Type of Analgesia						Mode of Delivery										
Author and Year	Opioid	LA	Paracetamol	NSAIDs	Holistic and Complementary	Other	Oral	Parenteral	Regional	Neuraxial	Local Infiltration	Intraperitoneal	IM	PC	Topical	Rectal	Other
																	
Abd-Elsalam 2017 [24]		x				x (Magnesium)			x								
Abdullah 2022 [20]		x							x								
Ariyasriwatana 2022 [37]	x			x	x (Curcuminoids)		x										
Chandveettil 2021 [25]	x	x	x							x				x			
Chantawong 2021 [26]	x			x		x (Abdominal binder)	x	x						x			x (Abdominal binder)
Cho 2021 [27]	x			x		x (Dexmedetomidine)		x						x			
Dang 2020 [38]	x							x						x			
Dong 2021 [8]	x	x		x				x			x			x			
Dong 2022 [9]	x			x													
El Hachem 2015 [39]	x	x	x	x			x	x	x								
Ferguson 2009 [40]	x	x		x				x		x				x			
Gottschalk 2002 [21]	x	x						x		x							
Gu 2017 [10]		x						x	x								
Güngördük2023 [41]		x				No analgesia stated		x							x		
Guo 2022 [42]	x			x				x						x			
Hayden 2020 [22]	x	x						x		x		x		x			
Hou 2021 [11]					x (Chinese medicine)	x (Psychological care)	x										x (Psychological care)
Jones 2000 [28]	x	x		x				x		x				x			
Kara 2012 [43]	x							x		x				x			
Kjølhede 2019 [44]	x	x	x	x			x	x		x	x						
Kuniyoshi 2021 [29]	x	x						x	x	x				x			
Lam 2022 [45]	x		x	x	x (Electro-acupuncture, auricular acupuncture)			x						x			
Liu 2018 [12]	x			x				x						x			
Lotfy 2022 [46]	x	x				x (Dexmedetomidine)		x		x							
Ma 2015 [13]	x			x				x									
Moslemi 2015 [30]	x	x						x		x							
Nong 2013 [47]	x			x				x						x			
Palaia 2022 [48]						x (Diet)	x										x (No standard of care)
Pearl 2002 [49]*	x		x	x			x	x						x			
Samulak 2011 [31]	x		x	x		x (Metamizol)		x			x					x	
Sattari 2020 [50]	x		x			x (Duloxetine)	x	x									
Shi 2021 [14]						x (Nursing)											x
Standl 2003 [23]	x	x						x		x				x			
Sugihara 2018 [51]	x	x	x	x							x					x	
Taylor 2003 [32]	x				x (Swedish massage/vibration)									x			
Tuncer 2003 [33]	x			x				x						x			
Ulm 2018 [52]	x		x	x		x (Gabapentin)	x	x						x			
Wang 2020 [15]	x					x (Ketamine)		x						x			
Wang 2016 [34]	x					x (Dexmedetomidine)		x					x	x			
Xia 2022 [35]				x	x Ultrasonic physiotherapy	x (Exercise, opioid receptor antagonist, abdominal girdle)		x									x (Exercise, abdominal girdle)
Yeh 2009 [53]	x					x (Naloxone)		x						x			
Zhou 2023 [16]	x			x				x	x					x			
Zhu 2021 [19]	x			x				x									
Zhu 2019 [17]	x							x						x			
Zhu 2024 [18]						x (Empathetic care, high protein diet)											x
Yazici 2021 [36]	x		x	x				x		x				x			

Abbreviations: LA = local anesthesia, NSAIDs = nonsteroidal anti-inflammatory drugs, IM = intramuscular, PC = patient controlled.

**Table 4:** Summary of outcomes reviewed in the studies. They were categorized according to pain, analgesia consumption, quality of life, physical function, psychological function, satisfaction, physiologic response, adverse events, long term, and procedural or operative outcomes.

Author and Year	Pain	Analgesia Consumption	Quality of Life	Physical Function	Psychological Function	Satisfaction	Physiologic Response	Adverse Events	Long Term	Procedural/Operative
**Cervical cancer (n = 11)**										
Dong 2021 [8]	x		x	x				x		x
Dong 2022 [9]	x				x		x	x		
Gu 2017 [10]	x	x	x					x		x
Hou 2021 [11]	x		x		x		x		x	
Liu 2018 [12]	x	x		x			x	x		
Ma 2015 [13]	x	x		x			x	x		x
Shi 2021 [14]	x			x	x	x	x	x		x
Wang 2020 [15]	x	x			x	x	x	x		x
Zhou 2023 [16]	x	x		x		x		x		x
Zhu 2019 [17]	x	x		x	x	x		x		x
Zhu 2024 [18]	x		x	x	x					
**Endometrial Cancer (n = 1)**										
Zhu 2021 [19]	x	x		x	x	x	x	x		x
**Ovarian Cancer (n = 4)**										
Abdullah 2022 [20]	x	x					x	x		x
Gottschalk 2002 [21]	x	x		x			x	x		x
Hayden 2020 [22]	x	x	x	x			x	x	x	x
Standl 2013 [23]	x	x		x				x	x	x
**Vulvar Cancer (n = 0)**										
**Generic Cancer (n = 13)**										
Abd-Elsalam 2017 [24]	x	x					x	x		x
Chandveettil 2021 [25]	x	x					x	x		
Chantawong 2021 [26]	x	x	x	x			x	x		x
Cho 2021 [27]	x	x		x	x			x	x	
Jones 2000 [28]	x	x					x	x		x
Kuniyoshi 2021 [29]	x	x					x	x		x
Moslemi 2015 [30]	x	x		x	x		x	x		x
Samulak 2011 [31]	x	x						x		x
Taylor 2003 [32]	x	x		x			x	x		x
Tuncer 2003 [33]	x	x				x		x		x
Wang 2016 [34]	x	x		x	x	x	x	x		x
Xia 2022 [35]	x						x	x		x
Yazici 2021 [36]	x	x		x			x	x		x
**Generic (Benign and Cancer) (n = 17)**										
Ariyasriwatana 2022 [37]	x							x		x
Dang 2020 [38]	x	x		x	x	x		x		
El Hachem 2015 [39]	x	x						x		x
Ferguson 2009 [40]	x	x			x	x	x	x		x
Güngördük 2023 [41]	x	x			x	x	x	x	x	x
Guo 2022 [42]	x	x		x	x	x	x	x		x
Kara 2012 [43]	x	x		x		x		x		
Kjølhede2019 [44]	x		x	x				x		x
Lam 2022 [45]	x	x	x	x		x		x		x
Lotfy 2022 [46]	x	x				x	x	x		
Nong 2013 [47]	x	x			x	x		x		x
Palaia 2022 [48]	x	x		x			x	x		x
Pearl 2002 [49]	x	x			x	x		x		x
Sattari 2020 [50]	x	x	x				x	x		x
Sugihara 2018 [51]	x	x					x	x		x
Ulm 2018 [52]	x	x		x			x	x		x
Yeh 2009 [53]	x	x						x		x

**Table 5:** Summary of journal impact, study funding, Jadad score and study size.

Author and Year	Publishing Journal Impact Factor	Study Funding Type	Jadad Score	Study Size
**Cervical (n = 11)**				
Dong 2021 [8]	4.06	N/A	2	98
Dong 2022 [9]	1.06	N/A	1	150
Gu 2017 [10]	3.111	N/A	3	62
Hou 2021 [11]	2.629	N/A	3	232
Liu 2018 [12]	4.996	N/A	5	56
Ma 2015 [13]	3.423	N/A	5	64
Shi 2021 [14]	4.06	N/A	3	324
Wang 2020 [15]	2.649	N/A	5	417
Zhou 2023 [16]	1.6	Unknown	5	154
Zhu 2019 [17]	1.817	N/A	5	83
Zhu 2024 [18]	1.329	None	5	196
**Endometrial Cancer (n = 1)**				
Zhu 2021 [19]	1.925	N/A	3	99
**Ovarian Cancer (n = 4)**				
Abdullah 2022 [20]	0.59	N/A	5	60
Gottschalk 2002 [21]	5.564	Yes	2	60
Hayden 2020 [22]	9.872	Government	5	40
Standl 2013 [23]	6.713	Yes	5	28
**Vulvar Cancer (n = 0)**				
**Generic Cancer (n = 13)**				
Abd-Elsalam 2017 [24]	4.965	N/A	5	60
Chandveettil 2021 [25]	5.77	N/A	3	60
Chantawong 2021 [26]	2.948	University	3	109
Cho 2021 [27]	4.468	N/A	5	91
Jones 2000 [28]	0.54	Yes	5	30
Kuniyoshi 2021 [29]	2.10	N/A	5	73
Moslemi 2015 [30]	3.56	University	3	90
Samulak 2011 [31]	0.196	N/A	1	128
Taylor 2003 [32]	2.381	Government	2	105
Tuncer 2003 [33]	0.196	N/A	4	50
Wang 2016 [34]	1.817	University	5	36
Xia 2022 [35]	0.4	Government	3	156
Yazici 2021 [36]	2.86	N/A	3	75
**Generic (Benign and Cancer) (n = 17)**				
Ariyasriwatana 2022 [37]	2.381	N/A	2	98
Dang 2020 [38]	2.832	N/A	5	124
El Hachem 2015 [39]	5.93	N/A	5	88
Ferguson 2009 [40]	5.482	N/A	2	135
Güngördük 2023 [41]	2.4	University	3	244
Guo 2022 [42]	2.217	N/A	5	112
Kara 2012 [43]	1.671	N/A	5	56
Kjølhede2019 [44]	3.007	University	3	77
Lam 2022 [45]	3.610	Research funding and company	5	72
Lotfy 2022 [46]	0.239	N/A	5	90
Nong 2013 [47]	2.24	N/A	5	79
Palaia 2022 [48]	2.1	N/A	3	168
Pearl 2002 [49]	3.90	N/A	3	120
Sattari 2020 [50]	0.35	N/A	5	60
Sugihara 2018 [51]	3.239	Research grant	3	194
Ulm 2018 [52]	5.482	N/A	3	138
Yeh 2009 [53]	3.282	N/A	4	169

## Data Availability

No new data were created or analyzed in this study. Data sharing is not applicable to this article.

## Supplementary Materials

The following supporting information can be downloaded at: https://www.mdpi.com/article/10.3390/cancers17162718/s1, Table S1: Electronic literature search strategies and Table S2: Risk of bias.

## Abbreviations

The following abbreviations are used in this manuscript:

BMI	Body Mass Index
CEA	Continuous Epidural Analgesia
CENTRAL	Cochrane Central Register of Controlled Trials
CRBS	Continuous Rectus Sheath Block
ESPB	Erector Spinae Plane Block
HS	Initials of Reviewer/Author
IQR	Interquartile Range
IV	Intravenous
MIS	Minimally Invasive Surgery
NMDA	N-methyl-D-aspartate (as in NMDA receptor)
NRS	Numerical Rating Scale
NSAIDs	Non-Steroidal Anti-Inflammatory Drugs
PCA	Patient-Controlled Analgesia
PCEA	Patient-Controlled Epidural Analgesia
PACU	Post-Anesthesia Care Unit
PRISMA	Preferred Reporting Items for Systematic Reviews and Meta-Analyses
RCT(s)	Randomized Controlled Trial(s)
SPSS	Statistical Package for the Social Sciences
SS	Initials of Reviewer/Author
TAP/TAPB	Transversus Abdominis Plane/Transversus Abdominis Plane Block
USA	United States of America
US	Ultrasound
VAS	Visual Analog Scale
