# Supplementary material for: Postoperative Pain Following Gynecology Oncological Surgery: A Systematic Review by Tumor Site
Source: Cancers (Basel). 2025 Aug 21;17(16):2718. doi: 10.3390/cancers17162718 (PMC12384201; doi:10.3390/cancers17162718)
Supplement: Supplementary file 1 [file cancers-17-02718-s001.zip › cancers-3782484-supplementary.pdf]

Supplementary Materials: Postoperative Pain following Gynecology Oncological Surgery: A Systematic Review by Tumor Site

Selina Chiu, Helen Staley, Xiaoxi Zhang, Anita Mitra, Flavia Sorbi, James Richard Smith, Joseph Yazbek, Sadaf Ghaem-Maghami, Sanooj Soni, Christina Fotopoulou and Srdjan Saso

Table S1. Electronic literature search strategies.

	**CENTRAL Search Strategy**
#1	MeSH descriptor Analgesia explode all trees
#2	MeSH descriptor Anesthetics explode all trees
#3	MeSH descriptor Analgesics explode all trees
#4	MeSH descriptor Pain explode all trees
#5	analgesia or analgesic*
#6	anesthetic* or anaesthetic*
#7	pain next/3 (relief or drug* or therap* or treat*)
#8	(#1 OR #2 OR #3 OR #4 OR #5 OR #6 OR #7)
#9	MeSH descriptor Postoperative pain explode all trees
#10	MeSH descriptor Postoperative Care explode all trees
#11	MeSH descriptor Postoperative Complications this term only
#12	((post operative or postoperative or post surg* or postsurg* or follow* surg* or follow* operat* or after surg* or after operat*) near (pain* or recovery or relief or drug* or therap* or treat* or analgesi*)):ti,ab
#13	#9 or #10 or #11 or #12
#14	#8 and #13
#15	MeSH descriptor Endometrial Neoplasms explode all trees
#16	endometr* near/5 (cancer* or tumor* or tumour* or neoplas* or malignan* or carcinom* or adenocarcinom*)
#17	(uter* and lining and (cancer* or tumor* or tumour* or neoplas* or malignan* or carcinom* or adenocarcinom*))
#18	(uter* near/5 (cancer* or tumor* or tumour* or neoplas* or malignan* or carcinom* or adenocarcinom*))
#19	#15 or #16 or #17 or #18
#20	#14 and #19
#21	MeSH descriptor Uterine Cervical Neoplasms explode all trees
#22	(cervi* near/5 (cancer* or neoplas* or tumor* or tumour* or malignan* or carcinoma* or adenocarcinoma*))
#23	#21 or #22
#24	#14 and #23
#25	MeSH descriptor Vulvar Neoplasms explode all trees
#26	vulva* adj5 (cancer* or carcinom* or tumor* or tumour* or malignan* or neoplasm*)
#27	#25 or #26
#28	#14 and #27
#29	ovar* and (cancer* or carcinom* or neoplasm* or tumor* or tumour* or malignan*)
#30	MeSH descriptor Ovarian Neoplasms explode all trees
#31	MeSH descriptor Fallopian Tube Neoplasms explode all trees
#32	MeSH descriptor Peritoneal Neoplasms explode all trees
#33	#29 or #30 or #31 or #32
#34	#14 and #33
	**PubMed search strategy**
#1	Analgesia[MeSH Terms]
#2	Anesthetics[MeSH Terms]
#3	Analgesics[MeSH Terms]
#4	Pain[MeSH Terms]
#5	(pain[tw] AND (relief[tw] OR drug*[tw] OR therap*[tw] OR treat*[tw]))
#6	(analgesia[tw] OR analgesic*[tw])
#7	(anesthetic*[tw] OR anaesthetic*[tw])

#8	#1 OR #2 OR #3 OR #4 OR #5 OR #6 OR #7
#9	(ovar*[tw] AND (cancer*[tw] OR tumor*[tw] OR tumour*[tw] OR neoplas*[tw] OR carcinoma*[tw] OR malignan*[tw]))
#10	Ovarian Neoplasms[MeSH Terms]
#11	Fallopian Tube Neoplasms[MeSH Terms]
#12	Peritoneal Neoplasms[MeSH Terms]
#13	#9 OR #10 OR #11 OR #12
#14	(vulv*[tw] AND (cancer*[tw] OR tumor*[tw] OR tumour*[tw] OR neoplas*[tw] OR carcinoma*[tw] OR malignan*[tw]))
#15	Vulvar Neoplasms[MeSH Terms]
#16	#14 OR #15
#17	(endometr*[tw] AND (cancer*[tw] OR tumor*[tw] OR tumour*[tw] OR neoplas*[tw] OR malignan*[tw] OR carcinom*[tw] OR adenocarcinom*[tw]))
#18	(uter*[tw] AND lining[tw] AND (cancer*[tw] OR tumor*[tw] OR tumour*[tw] OR neoplas*[tw] OR malignan*[tw] OR carcinom*[tw] OR adenocarcinom*[tw]))
#19	Endometrial Neoplasms[MeSH Terms]
#20	(uter*[tw] AND (cancer*[tw] OR tumor*[tw] OR tumour*[tw] OR neoplas*[tw] OR malignan*[tw] OR carcinom*[tw] OR adenocarcinom*[tw]))
#21	#17 OR #18 OR #19 OR #20
#22	(cervi*[tw] AND (cancer*[tw] OR tumor*[tw] OR tumour*[tw] OR neoplas*[tw] OR malignan*[tw] OR carcinom*[tw] OR adenocarcinom*[tw]))
#23	Uterine Cervical Neoplasms[MeSH Terms]
#24	#22 OR #23
#25	pain, postoperative[MeSH Terms]
#26	postoperative care[MeSH Terms]
#27	postoperative complication[MeSH Terms]
#28	(("post operative"[tiab] OR postoperative[tiab] OR post surg*[tiab] OR postsurg*[tiab] OR follow* surg*[tiab] OR follow* operat*[tiab] OR after surg*[tiab] OR after operat*[tiab]) AND (pain*[tiab] OR recovery[tiab] OR relief[tiab] OR drug*[tiab] OR therap*[tiab] OR treat*[tiab] or analgesi*[tiab]))
#29	#25 OR #26 OR #27 OR #28
#30	#8 AND #13 AND #29
#31	#8 AND #16 AND #29
#32	#8 AND #21 AND #29
#33	#8 AND #24 AND #29
	**Embase search strategy**
1	exp analgesia/
2	exp anesthetic agent/
3	exp analgesic agent/
4	exp pain/dt, th [Drug Therapy, Therapy]
5	(pain adj3 (relief or drug* or therap* or treat*)).mp.
6	(analgesia or analgesic*).mp.
7	(anesthetic* or anaesthetic*).mp.
8	1 or 2 or 3 or 4 or 5 or 6 or 7
9	exp ovary tumor/
10	(ovar* adj5 (cancer* or tumor* or tumour* or malignan* or neoplas* or carcinoma*)).mp.
11	exp Fallopian Tube Neoplasms/
12	exp Peritoneal Neoplasms/
13	9 or 10 or 11 or 12
14	exp vulva tumor/
15	(vulva* adj5 (cancer* or tumor* or tumour* or neoplas* or carcinoma* or malignan*)).mp.
16	14 or 15
17	exp endometrium tumor/
18	(endometr* adj5 (cancer* or tumor* or tumour* or neoplas* or malignan* or carcinom* or adenocarcinom*)).mp.
19	(uter* and lining and (cancer* or tumor* or tumour* or neoplas* or malignan* or carcinom* or adenocarcinom*)).mp.
20	(uter* adj5 (cancer* or tumor* or tumour* or neoplas* or malignan* or carcinom* or adenocarcinom*)).mp.
21	17 or 18 or 19 or 20
22	exp uterine cervix tumor/
23	(cervi* adj5 (cancer* or tumor* or tumour* or neoplas* or carcinoma* or malignan*)).mp.
24	22 or 23

25	exp Postoperative pain/
26	exp Postoperative Care/
27	postoperative complication/dt, pc, rh
28	pain/pc
29	((post operative or postoperative or post surg* or postsurg* or follow* surg* or follow* operat* or after surg* or after operat*) adj6 (pain* or recovery or relief or drug* or therap* or treat* or analgesi*)).ti,ab.
30	25 or 26 or 27 or 28 or 29
31	8 and 13 and 30
32	8 and 16 and 30
33	8 and 21 and 30
34	8 and 24 and 30
	**MEDLINE search strategy**
1	exp Postoperative complications/
2	Postoperative Complications/
3	exp Pain/
4	pain/
5	exp Analgesia/
6	exp Anesthetics/
7	exp Analgesics/
8	exp Pain/dt, th [Drug Therapy, Therapy]
9	(pain adj3 (relief or drug* or therap* or treat*)).mp.
10	(analgesia or analgesic*).mp.
11	(anesthetic* or anaesthetic*).mp.
12	5 or 6 or 7 or 8 or 9 or 10 or 11
13	(ovar* adj5 (cancer* or tumor* or tumour* or neoplas* or carcinoma* or malignan*)).mp.
14	exp Ovarian Neoplasms/
15	exp Fallopian Tube Neoplasms/
16	exp Peritoneal Neoplasms/
17	13 or 14 or 15 or 16
18	(vulv* adj5 (cancer* or tumor* or tumour* or neoplas* or carcinoma* or malignan*)).mp.
19	exp Vulvar Neoplasms/
20	18 or 19
21	(endometr* adj5 (cancer* or tumor* or tumour* or neoplas* or malignan* or carcinom* or adenocarcinom*)).mp.
22	(uter* and lining and (cancer* or tumor* or tumour* or neoplas* or malignan* or carcinom* or adenocarcinom*)).mp.
23	exp Endometrial Neoplasms/
24	(uter* adj5 (cancer* or tumor* or tumour* or neoplas* or malignan* or carcinom* or adenocarcinom*)).mp.
25	21 or 22 or 23 or 24
26	(cervi* adj5 (cancer* or tumor* or tumour* or neoplas* or malignan* or carcinom* or adenocarcinom*)).mp.
27	exp Uterine Cervical Neoplasms/
28	26 or 27
29	exp Pain, Postoperative/
30	exp Postoperative Care/
31	Postoperative complications/
32	Pain/
33	((post operative or postoperative or post surg* or postsurg* or follow* surg* or follow* operat* or after surg* or after operat*) adj6 (pain* or recovery or relief or drug* or therap* or treat* or analgesi*)).ti,ab.
34	29 or 30 or 31 or 32 or 33
35	12 and 17 and 34
36	12 and 20 and 34
37	12 and 25 and 34
38	12 and 28 and 34

Table S2. Risk of bias was evaluated across six domains using the Cochrane Collaboration's Risk of Bias tool: random sequence generation (selection bias), allocation concealment (selection bias), blinding of participants and personnel (performance bias), blinding of outcome assessment (detection bias), incomplete outcome data (attrition bias), selective reporting (reporting bias), and other bias. Trials are categorized into three groups: tumor-specific gynecological cancers, generic cancers, and generic (benign and cancers) studies. The graph represents the number of studies rated as low risk (green), some concerns (yellow), or high risk (red) in each domain.

	Random sequence generation (selection bias)	Allocation concealment (selection bias)	Blinding (performance bias and detection bias): all outcome	Incomplete outcome data (attrition bias): all outcome	Selective reporting (reporting bias)	Other bias
**Cervical (n = 11)**						
Dong 2021 [9]	yellow	yellow	yellow	green	yellow	yellow
Dong 2022 [11]	yellow	yellow	yellow	green	yellow	yellow
Gu 2017 [12]	green	yellow	yellow	green	yellow	yellow
Hou 2021 [13]	green	red	red	green	yellow	yellow
Liu 2018 [14]	green	green	green	green	yellow	yellow
Ma 2015 [15]	green	green	green	green	yellow	yellow
Shi 2021 [16]	green	yellow	yellow	green	yellow	yellow
Wang 2020 [17]	green	green	green	green	yellow	yellow
Zhou 2023 [19]	green	green	green	green	yellow	yellow
Zhu 2019 [18]	green	green	green	green	yellow	yellow
Zhu 2024 [20]	green	yellow	yellow	green	yellow	yellow
**Endometrial cancer (n = 1)**						
Zhu 2021 [21]	green	green	green	green	yellow	yellow
**Ovarian cancer (n = 4)**						
Abdullah 2022 [25]	green	green	green	green	yellow	yellow
Gottschalk 2002 [22]	yellow	green	green	green	yellow	yellow
Hayden 2020 [23]	green	green	green	green	yellow	yellow
Standl 2013 [24]	green	green	green	green	yellow	yellow
**Vulvar cancer (n = 0)**						
**Generic cancer (n = 13)**						
Abd-Elsalam 2017 [26]	green	green	green	green	red	yellow
Chandveettil 2021 [27]	green	yellow	red	green	red	yellow
Chantawong 2021 [28]	green	green	red	green	red	yellow
Cho 2021 [37]	green	green	green	green	red	yellow
Jones 2000 [29]	green	green	green	green	red	yellow
Kuniyoshi 2021 [30]	green	yellow	red	red	red	yellow
Moslemi 2015 [32]	green	yellow	red	green	red	yellow
Samulak 2011 [33]	yellow	yellow	yellow	green	red	yellow
Taylor 2003 [34]	yellow	yellow	red	green	red	yellow
Tuncer 2003 [35]	yellow	green	green	green	red	yellow
Xia 2022 [38]	green	yellow	red	green	red	yellow
Yazici 2021 [31]	green	green	red	green	red	yellow
Wang 2016 [36]	green	green	green	green	red	yellow
**Generic (Benign and cancer) (n = 17)**						
Ariyasriwatana 2022 [39]	green	green	green	green	red	yellow
Dang 2020 [40]	green	green	green	green	red	yellow
El Hachem 2015 [41]	green	green	green	green	red	yellow
Ferguson 2009 [42]	green	red	red	green	red	yellow
Güngördük 2023 [54]	green	green	red	green	red	yellow
Guo 2022 [43]	green	green	green	green	red	yellow
Kara 2012 [44]	green	green	green	green	red	yellow
Kjølhede2019 [45]	green	red	red	green	red	yellow
Lam 2022 [46]	green	green	green	yellow	red	yellow
Lotfy 2022 [47]	green	green	green	green	red	yellow

Nong 2013 [48]						
Palaia 2022 [55]						
Pearl 2002 [49]						
Sattari 2020 [50]						
Sugihara 2018 [51]						
Ulm 2018 [52]						
Yeh 2009 [53]						
